# Genetic Background Shapes Phenotypic Response to Diet for Adiposity in the Collaborative Cross

**DOI:** 10.3389/fgene.2020.615012

**Published:** 2021-02-11

**Authors:** Phoebe Yam, Jody Albright, Melissa VerHague, Erik R. Gertz, Fernando Pardo-Manuel de Villena, Brian J. Bennett

**Affiliations:** ^1^Integrative Genetics and Genomics Graduate Group, University of California, Davis, Davis, CA, United States; ^2^Western Human Nutrition Research Center, Agricultural Research Service, US Department of Agriculture, Davis, CA, United States; ^3^Nutrition Research Institute, University of North Carolina, Chapel Hill, NC, United States; ^4^Department of Genetics, Lineberger Comprehensive Cancer Center, University of North Carolina, Chapel Hill, NC, United States; ^5^Department of Nutrition, University of California, Davis, Davis, CA, United States

**Keywords:** collaborative cross, diet, nutrigenomics and nutrigenetics, genetics, obesity

## Abstract

Defined as chronic excessive accumulation of adiposity, obesity results from long-term imbalance between energy intake and expenditure. The mechanisms behind how caloric imbalance occurs are complex and influenced by numerous biological and environmental factors, especially genetics, and diet. Population-based diet recommendations have had limited success partly due to the wide variation in physiological responses across individuals when they consume the same diet. Thus, it is necessary to broaden our understanding of how individual genetics and diet interact relative to the development of obesity for improving weight loss treatment. To determine how consumption of diets with different macronutrient composition alter adiposity and other obesity-related traits in a genetically diverse population, we analyzed body composition, metabolic rate, clinical blood chemistries, and circulating metabolites in 22 strains of mice from the Collaborative Cross (CC), a highly diverse recombinant inbred mouse population, before and after 8 weeks of feeding either a high protein or high fat high sucrose diet. At both baseline and post-diet, adiposity and other obesity-related traits exhibited a broad range of phenotypic variation based on CC strain; diet-induced changes in adiposity and other traits also depended largely on CC strain. In addition to estimating heritability at baseline, we also quantified the effect size of diet for each trait, which varied by trait and experimental diet. Our findings identified CC strains prone to developing obesity, demonstrate the genotypic and phenotypic diversity of the CC for studying complex traits, and highlight the importance of accounting for genetic differences when making dietary recommendations.

## Introduction

Obesity is a complex disease characterized by excessive adipose tissue accumulation and has become one of the leading preventable causes of death in both developed and developing countries (Bell et al., 2005; Friedman, 2015; WHO, 2015). Fundamentally, obesity results from a chronic imbalance between energy intake and expenditure (Hill et al., 2012; Romieu et al., 2017; Swift et al., 2018; Oussaada et al., 2019). This imbalance is caused by numerous biological factors including: genetics (Bell et al., 2005; Singh et al., 2017; Loos, 2018), metabolism (Timper and Brüning, 2017; Speakman, 2018; Fernández-Verdejo et al., 2019), and the gut microbiome (John and Mullin, 2016; Martinez et al., 2016; Torres-Fuentes et al., 2017), as well as environmental factors such as chemical exposure (Janesick and Blumberg, 2016; Heindel and Blumberg, 2019; Shahnazaryan et al., 2019) and diet, particularly in the context of overfeeding relative to physical activity levels (Sims, 1976; Danforth, 1985; Schmidt et al., 2012; Cuthbertson et al., 2017; Creasy et al., 2018).

Identification of the underlying genes predisposing an individual to obesity has been a very active area of investigation. Large-scale human genome-wide association studies (GWAS) that test the association of millions of genetic variants with adiposity, body mass index, and waist-to-hip ratio have identified >300 genetic loci for obesity traits, such as the *FTO, TMEM18, CADM2*, and *LYPLAL1* loci, among others (Loos et al., 2008; González-Muniesa et al., 2017; Loos, 2018). Complementing approaches in humans, studies in mice have provided fundamental insights into the genetic regulation of adiposity and susceptibility to obesity (Coleman and Hummel, 1974; Lu et al., 1994; Carroll et al., 2004; Attie et al., 2017). For example, the genes that encode leptin and leptin receptor which arose as spontaneous deficiency mutations in *ob/ob* and *db/db* obese mice respectively (Ingalls et al., 1950; Hummel et al., 1966) were shown to regulate satiety after gene cloning was possible (Zhang et al., 1994; Tartaglia et al., 1995). Similarly, the link between the *FTO* gene and obesity was first reported in mice prior to the identification of this gene's association with obesity in humans (Fischer et al., 2008). The similar biology between humans and mice in terms of physiology, morphology, and genetics, and the ability to manipulate the mouse genome has aided our understanding of the underlying mechanisms affecting energy balance and obesity (Robinson et al., 2000; Pomp et al., 2008).

Similarly, diet is among the most studied environmental factors, as it remains an important and potentially successful focus of public health interventions (Wilborn et al., 2005; Eknoyan, 2006; Makris and Foster, 2011). One of the difficulties identifying the optimal dietary recommendation for a population is the inter-individual variation observed in response to diet (Berry et al., 2020). At a certain level there may be no “perfect” diet that works universally across populations to mitigate obesity (Dansinger et al., 2005; Johnston et al., 2014). Thus, in spite of the successes of GWAS and dietary intervention studies, there still remains practical public health challenges for understanding and preventing obesity. Animal models often solve some of the challenges by limiting confounding environmental influences to gain a more complete understanding of the etiology of obesity. Studies performed using inbred mouse strains suggest that phenotypic response to diet occurs in a strain-dependent manner (West et al., 1992, 1995; Barrington et al., 2017). Understanding the interaction of genetics and diet offers insight into how “precision nutrition” could improve and refine our dietary recommendations.

In order to broaden our understanding of how genetics and diet impact obesity at both the individual and population levels in a genetically diverse population, we analyzed how consumption of diets with different macronutrient compositions altered adiposity and other physiological traits in 22 strains of mice from the Collaborative Cross (CC), a large recombinant inbred mouse population generated from elaborate intercrosses of C57BL/6J, A/J, NOD/ShiLtJ, NZO/HiLtJ, 129S1/SvImJ, WSB/EiJ, CAST/EiJ, and PWK/PhJ, mouse strains (Churchill et al., 2004; Iraqi et al., 2008; Threadgill and Churchill, 2012). The tremendous genetic diversity of the CC population (Philip et al., 2011; Collaborative Cross Consortium, 2012; Srivastava et al., 2017; Shorter et al., 2019) facilitates the discernment between effects caused by diet from effects caused by genetic variation when measuring differences and changes in adiposity and other metabolic traits across multiple genetic “replicates” in each strain, thereby increasing power, reproducibility, and relevance to obesity in humans (Mathes et al., 2011). Following a 2-week acclimation period on standard synthetic diet (AIN-76A) to determine baseline phenotypes, mice between 8 and 11 weeks of age were randomized and put on experimental diets (high fat high sucrose or high protein) for 8 weeks, followed by analysis of body composition, metabolic rate, clinical blood chemistries, and circulating metabolites to assess the effect of diet on each trait since diets with higher protein, low glycemic index, and lower fat content may assist in maintaining weight loss compared to diets with higher carbohydrate content (Abete et al., 2010; Larsen et al., 2010; Hu et al., 2018; Myrmel et al., 2019; San-Cristobal et al., 2020). While both genetics and diet interact to influence adiposity and other phenotypes, health outcomes were more strongly impacted by genetic effects than diet. Furthermore, the effect of diet on each trait varied depending on CC strain, indicating that genetics determine how a particular diet may affect body composition.

## Materials and Methods

### Animals and Husbandry

**Figure 1:**
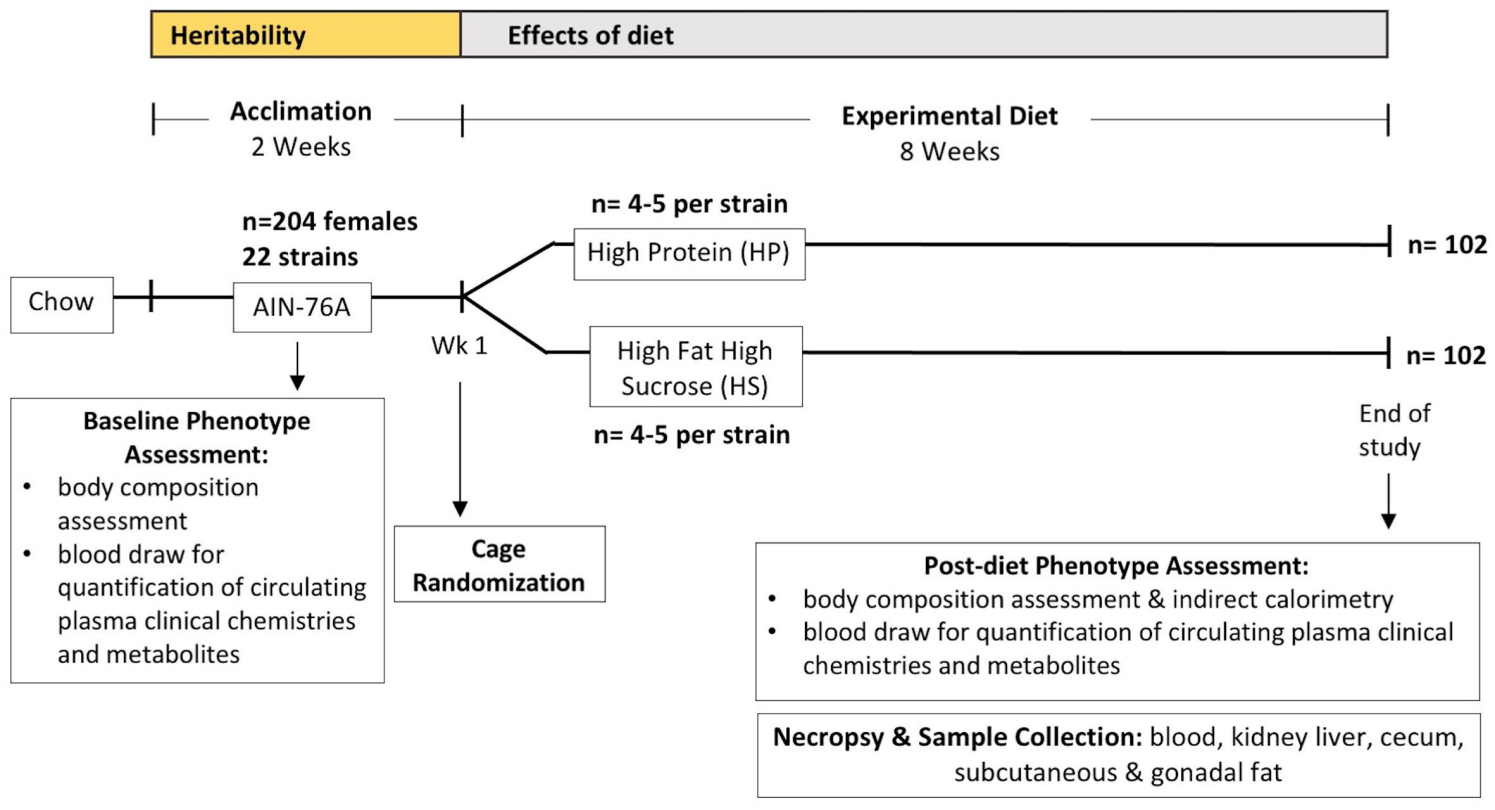
Experimental design and timeline. Collaborative Cross (CC) mice were obtained between 6 and 9 weeks of age (*n* = 204) and acclimated for 2 weeks on standard synthetic diet (AIN-76A) for baseline phenotype assessment which included body composition assessment and a blood draw for quantification of circulating plasma clinical chemistries and metabolites before cage randomization and starting diet challenges on either high protein (HP) or high fat high sucrose (HS) diet between 8 and 11 weeks of age, with an average age of 9.4 weeks. For each CC strain, 4–5 mice were assigned to each experimental diet except for CC024/GeniUnc which had 2 mice assigned to each experimental diet; the number of mice from each strain assigned to each diet are shown in Supplementary Table 1. Mice were subsequently maintained on experimental diets for a total of 8 weeks, with the final phenotype assessment performed the following week (week 9) which included another body composition assessment and indirect calorimetry to measure metabolic rate and activity. Samples collected during the necropsy were blood used in the current study, kidney, liver, subcutaneous and gonadal fat, and cecum samples for additional studies.

Female mice from 22 CC strains were obtained in 2016 from University of North Carolina's Systems Genetics Core Facility (Welsh et al., 2012) (total *n* = 204, Figure 1). All strains used are listed in Supplementary Table 1. Mice were then acclimated for 2 weeks on standard synthetic diet (AIN-76A), housed three mice per cage at 22°C with non-irradiated pine bedding, and provided free access to sterile water in a climate-controlled facility under a 12-h light/dark cycle. Mice were put on experimental diets between 8 and 11 weeks of age after the 2-week acclimation period, randomized into different cages by experimental diet (Supplementary Figure 1, Supplementary Table 1), and housed under the same conditions. After randomization, mice were challenged on their respective diets for 8 weeks, and analysis of body composition, metabolic rate, and physical activity were performed at the UNC Animal Metabolism Phenotyping Core post diet challenge (methods for analysis of body composition, metabolic rate, and physical activity described below) followed by necropsy and tissue collection. Because only a limited number of mice were available at one time, experiments spanning 11 weeks (2 weeks of acclimation, 8 weeks of diet challenge, post-diet phenotype assessments) for each “batch” were performed in 7 batches, where each batch contained about 33 mice on average, except for batch 6 which contained 14 mice. All mice were maintained on their respective experimental diets for the remainder of the study using protocols in accordance with the University of North Carolina Institution Animal Care and Use Committee guidelines. All maintenance protocols and experimental procedures were approved by the IACUC at University of North Carolina (UNC) Chapel Hill (IACUC Protocol Number: 13-103).

### Diets

During the 2-week acclimation period, mice were maintained on the defined synthetic diet containing 20.8% kcal protein, 67.7% kcal carbohydrate, and 11.5% kcal fat, referred to as AIN-76A in this study (D10001, Research Diets, New Brunswick, NJ; Supplementary Table 2) until 8–11 weeks of age to account for differences due to variable components of standard chow. Subsequently, one sibling from each of the 102 sibling trios was randomly assigned to each experimental diet (Supplementary Table 1). One hundred and two mice were transferred to a synthetic high fat high sucrose diet (HS) containing 16.8% kcal protein, 51.4% kcal carbohydrate, and 31.8% kcal fat, and 102 mice were placed on a high protein diet (HP) containing 40% kcal protein, 40% kcal carbohydrate and 20% kcal fat (D12266B and D12083101, respectively, Supplementary Table 2; Research Diets, New Brunswick, NJ).

### Body Composition and Weight

Body composition (lean and fat mass) was assessed in all cohorts during the first week of the acclimation phase to establish baseline phenotypes, as well as after 8 weeks of the experimental diet challenge using the Echo MRI-130 Body Composition Analyzer (EchoMRI, Houston, TX, USA). Body fat and lean mass percentages were calculated by dividing fat mass by scale weight and dividing lean mass by scale weight, respectively.

### Metabolic Rate and Activity

Mice were placed into individual indirect calorimetry cages (Phenomaster, TSE SYSTEMS, Chesterfield, MO) the week immediately following the 8 weeks of the experimental diet challenge for ~3 days and two nights (~48 h) to obtain O_2_ consumption and CO_2_ production, activity, and feed and water consumption measurements. After an 8-h acclimation period, data were collected for two complete 12-h night cycles and one complete 12-h day cycle every 42 min (Supplementary Figure 2). Basal activity was measured in three dimensions (x, y, and z) as breaks in the two infrared light beam frames that surrounded each cage. Rearing was detected by beam breaks in the z axis and total physical activity was defined as the sum of beam breaks in all three axes in counts. Feed and water were available *ad libitum* and consumption was measured by weighing sensors that held containers for feed and water, respectively, and recorded the amount of feed or water consumed. Spilled feed and water were caught by extended attachments on the feed and water containers suspended from the weighing sensors, so spilled feed and water were not recorded as consumed.

Heat production calculations were performed two ways by the TSE software (LabMaster) using O_2_ consumption and CO_2_ production measurements: (1) for the computation of total body weight (kcal/h/kg), and (2) for the computation of an exponent lean body mass assigned to total body weight (kcal/h/kg). From the exported raw data, energy consumption was calculated by multiplying feed consumption measurement (in grams) by the calorie (kcal) content per gram feed for each diet (Supplementary Table 2). Protein, carbohydrate, and fat consumption were calculated by multiplying the feed consumption measurement (in grams) by macronutrient content (in grams) per total gram of feed. For example, the average protein consumption for mice on the high protein diet was calculated by multiplying the measured feed consumed (g) by (40.6 g protein/90.3 g feed total).

Individual and combined diurnal means were calculated for each metabolic measurement using data collected at time points between the start and end times of the day cycle. Likewise, individual and combined nocturnal means were calculated for each metabolic measurement using data collected at time points between the start and end times of the night cycle (Supplementary Figure 2). Means for each measure were also calculated by date, e.g., mean of feed consumption for both light and dark cycles on the second day of the experiment.

### Biological Samples Collection

Tail clippings and blood samples were collected immediately before putting mice on experimental diets to establish baseline values. To collect tail clippings, tail tips were cleaned with 70% ethanol, and up to 5 mm of the tail tips were excised with sterile scissors and placed in 2 ml screw-cap tubes. After 8 weeks on experimental diets, mice were anesthetized via isoflurane inhalation and euthanized using cervical dislocation during the necropsy following a 4-h fast. Blood, kidney, liver, subcutaneous and gonadal fat, and cecum samples were collected (Figure 1). Blood samples were collected via retro-orbital bleed with heparinized capillary tubes into EDTA tubes, placed on ice, and centrifuged at 6,000 rpm for 10 min at 4°C for plasma collection. Plasma was then transferred to 1.5 ml Eppendorf tubes. Tissues were placed in 2 ml screw-cap tubes and snap frozen in liquid nitrogen. All plasma, frozen tissues, and previously collected samples were stored at −80°C. Additional gonadal fat, kidney, and liver tissues were fixed in capped glass vials containing 10% formalin and stored at room temperature.

### Plasma Clinical Chemistries

Cholesterol, triglyceride (TG), glucose, albumin, creatinine, urea, aspartate transaminase (AST), and alanine transaminase (ALT) levels were quantified using the Cobas Integra 400 Plus (Roche Diagnostics, Indianapolis, IN), according to manufacturer's instructions. An internal control (Human UTAK) was used to assess run variation. Baseline and post-diet circulating insulin were measured using ultrasensitive mouse insulin ELISA (ALPCO Diagnostics, Salem, NH) per manufacturer's instructions except for the following adjustment: 15 μl of plasma sample dilutions were used in the assay and back calculations were performed to determine actual plasma concentrations. Insulin optical density (OD) was measured at 450 nm using a spectrophotometric BioTek Synergy 2 plate reader (BioTek Instruments Inc, Winooski, VT). Insulin concentrations were derived from measured ODs using BioTek's Gen5 software.

### Liquid Chromatography-Mass Spectrometry (LC-MS)

Baseline and post-diet circulating trimethylamine N-oxide (TMAO), choline, phosphocholine, betaine, and carnitine were quantified using liquid chromatography–mass spectrometry (LC-MS) methods described by Wang et al. (2014) with modifications. Standards ranging from 0 to 100 μM of non-deuterated analytes in methanol were run in order to establish analyte standard curves. Two-fold serial dilutions of a 100 μM stock solution in methanol was used to make 13 standards. 5 μM of surrogate standard (SSTD) were prepared comprising of deuterated analytes in methanol. All standards were purchased from Sigma-Aldrich (St. Louis, MO). All reagent solvents were mass spectrometry grade and purchased from Fisher Scientific (Waltham, MA). Details of the protocol are contained in the data supplement and Supplementary Table 3.

### Statistical Methods

#### Determining Contributors to Phenotypic Variance

All phenotype data were tested for normality using the Shapiro-Wilk test in the statistical programming language R (R Core Team, 2019). Baseline non-normal data were transformed using power transformation or rank normalization if necessary before linear fixed models were fitted using CC strain and mouse batch (“week” in Supplementary File 2) as fixed effects to test for significant CC strain effects on phenotypic variance. Post-diet non-normal data were also normalized using these methods as appropriate for fitting linear mixed models using restricted maximum likelihood (REML) to determine the significance of the effect of diet and strain/diet interactions. To test for the significance of the effect of diet underlying phenotypic variance, linear mixed model analysis of the relationship between diet and phenotypic traits was performed using R and packages *lme4* (Bates et al., 2015), *lmerTest* (Kuznetsova et al., 2017), and *car* (Fox and Weisberg, 2019) for each post-diet phenotype. For models testing diet as the main effect, fixed effects included experimental diet and mouse batch, and random effects (intercepts) included CC strain, CC strain × experimental diet, randomization cage nested within experimental diet, and baseline cage nested within CC strain. In models used to test for the significance of the effect of strain/diet interactions, linear mixed models were fit for each post-diet phenotype, which included CC strain, experimental diet, CC strain × experimental diet, and mouse batch as fixed effects, and randomization cage nested within experimental diet and baseline cage nested within CC strain as random effects (intercepts). Visual inspection of residual plots did not reveal obvious deviations from homoscedasticity or normality. *P*-values were obtained by implementing Satterthwaite approximations as described by Luke (2016).

#### Calculation of Health Scores to Estimate Overall Metabolic Health

Metabolic health scores were calculated for all mice at baseline and 9 weeks post-diet. First, Z scores were calculated for several metabolic risk factors (circulating glucose, insulin, glucose/insulin ratio, cholesterol, TG, and body fat %) measured at baseline and post-diet for each mouse; the distribution used to calculate the Z score for baseline was all baseline samples, while samples were separated by diet before calculating post-diet Z scores. Next, the Z scores for each metabolic risk factor were added together, and then multiplied by −1 so that decreased health is reflected by a lower health score.

#### Baseline Broad-Sense Heritability Estimates

From linear models fitted using baseline normalized data with CC strain and mouse batch as the covariates used to test for significant CC strain effects on phenotypic variance (described above), broad-sense heritability (H^2^) was estimated for each phenotype by calculating the intraclass correlation (r_I_) and the coefficient of genetic determination (g^2^) using derived values for mean square between (MSB) strains and mean square within (MSW) strains (Festing, 1979). r_I_ may be interpreted as the proportion of total phenotypic variation that is accounted for by differences between strains, while g^2^ accounts for the additive genetic variance that doubles during inbreeding (Festing, 1979; Falconer, 1989; Lightfoot et al., 2001), so g^2^ is a more appropriate estimate for broad sense heritability in this study. However, other studies sometimes only provide one estimate or the other, so we have included both values to facilitate comparisons with other findings in the literature. r_I_ and g^2^ were calculated using the following formulas, where *n* is the number of mice per strain:

$$\begin{array}{l} {\text{r}}_{\text{I}}=\frac{\left(\text{MSB}-\text{MSW}\right)}{\text{MSB}+\left(n-1\right)\text{MSW}}\;g^{2}=\frac{\left(\text{MSB}-\text{MSW}\right)}{\text{MSB}+\left(2n-1\right)\text{MSW}} \end{array}$$

The number of mice per strain varies in this study, so *n* was calculated as:

$$\begin{array}{l} n=\frac{1}{\left(a-1\right)}\left(N-\frac{\;\Sigma \;{n_{i}}^{2}}{N}\right) \end{array}$$

where a is the number of strains, n_*i*_ is the number of mice in the *i*th strain, and N is the total number of mice (samples) per phenotype.

#### Post-diet Broad-Sense Heritability Estimates

Post-diet broad-sense heritability estimates (H^2^) were calculated for each trait to contrast the proportion of relative heritable variation attributed to genetics or diet, and to assess whether different diet “environments” affect heritability. Post-diet intraclass correlation (r_I_) values and the coefficients of genetic determination (g^2^) were calculated using the formulas above and the MSB and MSW derived from four different linear models: (1) a “full” additive model with strain, diet, and week as variables fitted with phenotype data from both experimental diets, (2) a “partial” additive model including strain and week as variables (diet excluded) fitted with phenotype data from both experimental diets, (3) a “HP” additive model including strain and week as variables fitted with phenotype data from only mice fed the HP diet, and (4) a “HS” additive model including strain and week as variables fitted with phenotype data from only mice fed the HS diet. H^2^ estimates derived from models fitted with data from all mice post-diet compare the contribution of genetics (strain) and diet overall to heritable phenotypic variance, while diet-specific H^2^ estimates were calculated to discern differences in heritability affected by differences in macronutrient composition.

#### Quantification of Heritable Variation Attributed to Genetics, Diet, and Gene × Diet Interactions

Linear mixed models with strain, diet, and strain x diet interactions as random effects (intercepts) were fitted using all post-diet phenotype data for body fat % and obesity-related traits to quantify the relative heritable variation attributed to genetics, diet, and gene × diet interactions based on the variance of each term in the model. The approximate values for the proportion of variance for strain, diet, and interaction were calculated by dividing the variance for each term by the sum of the variance for all terms in the model (including residuals).

#### Quantification of Diet Effect Size

To quantify size effects of diet on each trait, Hedges' g values for the HP diet were calculated by using the baseline-specific (AIN-76A) mean of the phenotype minus the HP-specific mean of the phenotype (M_1_-M_2_), and then dividing this value by the weighted pooled standard deviation (SD) for the two groups (Ellis, 2009):

$$\begin{array}{l} \text{Hedge}\text{s}{\;}^{\prime }\;g=\frac{M_{1}-M_{2}}{SD{*}_{pooled}} \end{array}$$

The weighted pooled SDs was calculated using the following equation where n_1_ = the number of samples from mice on the AIN-76A diet and n_2_ = the number of samples from mice on the HP diet:

$$\begin{array}{l} S{D^{*}}_{pooled}=\;\sqrt{\frac{\left(n_{1}-1\right)S{D_{1}}^{2}+\left(n_{2}-1\right)S{D_{2}}^{2}}{n_{1}+n_{2}-2}} \end{array}$$

Calculations for Hedges' g were performed using the following function from the effsize package in R (Torchiano, 2019), with pooled weighted SD, unpaired samples, removed NA entries and a 95% confidence interval, where d = phenotype measurements and f = experimental diets: cohen.d (d, f, pooled = TRUE, paired = FALSE, na.rm = TRUE, hedges.correction = TRUE, conf.level = 0.95). Corrected Hedges' g effect sizes are presented as standard deviation units so that a Hedges' g value of 1 indicates that the baseline diet and respective experimental diet differ by 1 standard deviation, a g of 2 indicates they differ by 2 standard deviations, and so on with the sign indicating the direction of change between diets. Positive Hedges' g indicates increased phenotype values post-diet compared to baseline, e.g. body fat % was increased from baseline in mice after feeding them the HP diet. Magnitude descriptions are based on the following cut-offs of |g|: negligible <0.2 < small <0.5 < medium <0.8 < large. Hedges' g values were calculated for the HS diet for each trait using the same method.

To further quantify the effect size of diet, we also calculated the intraclass correlation (ICC) for diet using the mean square between (MSB) diets and mean square within (MSW) diets derived from post-diet linear models including strain, diet, and week as variables, using the following formula where n = number of mice on each diet:

$$\begin{array}{l} \text{ICC}=\frac{\left(\text{MSB}-\text{MSW}\right)}{\text{MSB}+\left(n-1\right)\text{MSW}} \end{array}$$

The ICC for diet can be interpreted as the proportion of the total phenotypic variation that is accounted for by differences between diet.

#### Testing Significance of Phenotypic Difference Between Day and Night Cycles for Metabolic Traits

Phenotype data for metabolic traits were viewed in histograms to check for normality of the distributions, revealing skewness and non-normality. Thus, Wilcoxon signed rank tests with continuity correction were performed instead of student's *t*-tests using the following function from the stats package in R (R Core Team, 2019), with paired samples and a 95% confidence interval, where day = diurnal metabolic trait data and night = corresponding nocturnal metabolic trait data: wilcox.test (day, night, paired = TRUE, conf.int = TRUE).

#### Additional Statistical Analyses

All statistical analyses were performed in R (R Core Team, 2019). Summary statistics were calculated for all phenotypic data, include means and standard error (SE). Spearman's correlations were performed to determine significant relationships between traits at baseline and post-diet. To ascertain the magnitude of the effect of diet behind gene x environmental effects found for each trait in our linear mixed models, Spearman's correlation analysis was performed between the F-statistic of the gene x diet interactions of our models and Hedges' effect size for both diets (|g|). Each trait was categorized as either largely affected by diet (|g| > 0.8) or not (|g| <0.8), and significantly affected by gene x diet interactions (*p* <0.05) or not (*p* > 0.05), followed by Chi square analysis of whether the effect size of diet and the gene x environment are significantly related for the given trait.

## Results

### Baseline Traits Show Extensive Phenotypic Variation Among CC Strains

**Figure 2:**
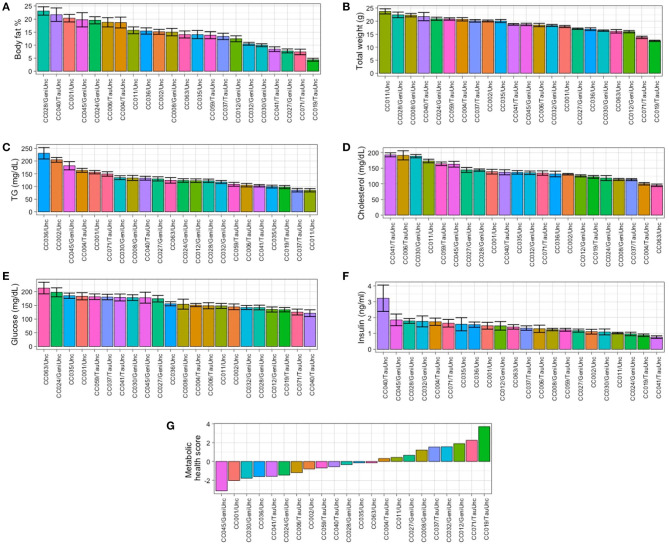
Body composition, circulating metabolic health marker levels, and metabolic health score are strain-dependent in the Collaborative Cross. At baseline, the Collaborative Cross demonstrates phenotypic variation in a strain-dependent manner. Baseline measurements of metabolic phenotypes are shown for **(A)** body fat %, **(B)** total weight, **(C)** triglycerides (TG), **(D)** total cholesterol, **(E)** glucose, **(F)** insulin, and **(G)** metabolic health score by strain during the 2-week acclimation period while mice were fed the baseline diet (AIN-76A). For metabolic health score **(G)**, strains are ordered from left to right by least healthy to most healthy. Data are mean ± SE for **(A–F)**; data are mean for **(G)**. For body fat % and total weight, 8-10 mice were available per strain, except for CC024/GeniUni (*n* = 4). For TG, cholesterol, glucose, insulin, and metabolic health score, 8–10 mice were available per strain, except for CC024/GeniUni (*n* = 4), and CC063/Unc (*n* = 6). Baseline linear models with CC strain and week as a covariate showed significant CC strain effects for all phenotypes shown (*p* <2.87 × 10^−6^).

Baseline values for adiposity (synonymous with body fat % in this study), clinical blood chemistries, and circulating metabolites were established to assess the degree of phenotypic variation due to genetic background of the CC strain (see Methods and Figure 1). Adiposity and circulating metabolic health marker levels exhibited wide ranges of phenotypic variation by CC strain (Figure 2) and there was a wide range of adiposity in the CC population ranging from 1.1 to 29.8% body fat, with strain CC019/TauUnc least susceptible to obesity (average body fat 4.4 ± 0.6%) and strain CC028/GeniUnc most susceptible (average body fat 23.1 ± 1.5%) (Figure 2A). Similarly, there was a wide range in average weight across the CC lines ranging from 12.4 ± 0.2 g in strain CC019/TauUnc to 23.7 ± 1.0 g in line CC011/Unc (Figure 2B). Within CC strains, CC040/TauUnc had the highest range of variability in adiposity (1.7–29.3%), while CC030/GeniUnc had the lowest range of variability in adiposity (7.6–12.6%). CC040/TauUnc had the highest variability in weight (11–28 g), while CC019/TauUnc had the lowest variability in weight (11.1–13.2 g). Linear regression analysis was performed to assess the significance of the effect of strain on each of the measured traits at baseline (Supplementary Table 4), and strain was found to have a significant effect on almost all traits, especially body fat % (*F* = 12.44, *p* = 7.71 ×10^−25^) and weight (*F* = 19.39, *p* = 3.95 × 10^−35^). To estimate the overall health of the mice from each CC strain, a metabolic health score was calculated using the sum of Z scores from measurements of several metabolic risk factors (circulating glucose, insulin, glucose/insulin ratio, cholesterol, TG, and body fat %). While the health score includes body fat % as one of the components, the phenotypes exhibited across CC strains for circulating analytes typically used as markers of metabolic health (circulating glucose, insulin, glucose/insulin ratio, cholesterol, TG) varied so that although one strain may have high body fat %, it may simultaneously have low levels of TG or glucose, such as CC040/TauUnc at baseline. Because metabolic health is determined by multiple phenotypes, the health score provides a way to estimate overall metabolic health for each CC strain in a way that accounts for these differences. For example, at baseline CC028/GeniUnc had the highest BF% but its health score was close to 0, so it was not exceedingly unhealthy relative to the other strains in this study despite its high BF%, since this strain's glucose, TG, and cholesterol levels were not elevated. Similar to adiposity and circulating analytes (Figures 2C–F), metabolic health also showed a wide range of phenotypic variation by CC strain at baseline (Figure 2G), where most strains with higher adiposity also appeared to have decreased metabolic health (Figures 2A,G), with the exception of CC028/GeniUnc.

### Total Body Weight Has a Limited Effect on Increased Adiposity

**Figure 3:**
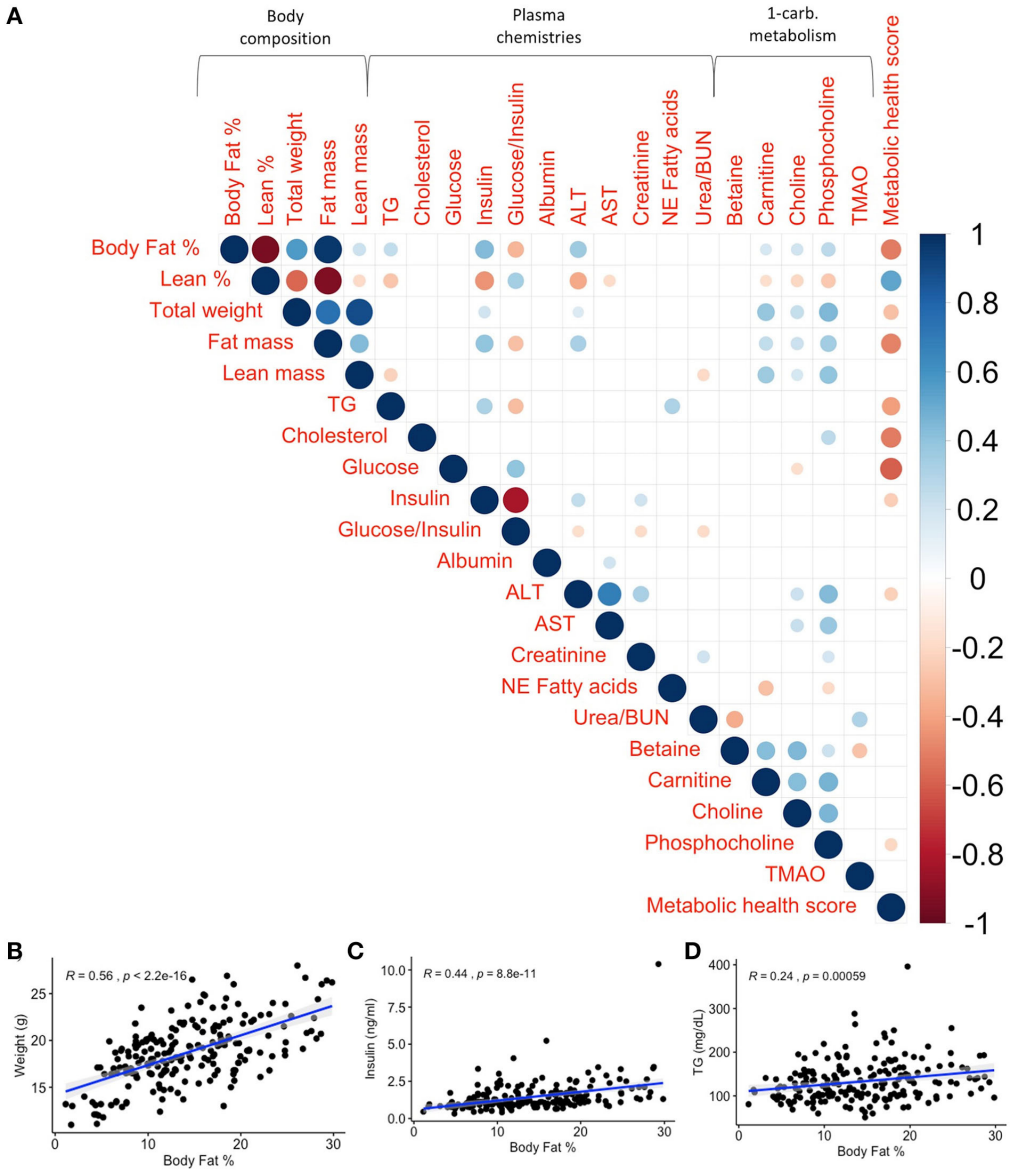
Phenotypic correlations at baseline illustrate the strength of relationships between traits without the influence of diet. Body fat % shows the strongest relationship with weight, insulin, triglycerides (TG), and metabolic health score at baseline compared to other traits. **(A)** Spearman's correlations of baseline phenotypes with p values adjusted using the Benjamini–Hochberg method. Only significant correlations (*p*_*adj*_ < 0.05) are shown. Scale indicates rho value. Spearman's correlations between baseline body fat % and **(B)** weight (*R* = 0.56, *p* <2.2 × 10^−16^), **(C)** insulin (*R* = 0.44, *p* = 8.8 × 10^−11^), and **(D)** TG (*R* = 0.24, *p* = 0.00059) show significant correlations between body fat % and obesity-associated phenotypes. *R* is Spearman's rho.

To determine whether total body weight predicts susceptibility to increased adiposity, body fat % was correlated with total body weight. Although the leanest strain overall (CC019/TauUnc, average body fat 4.41 ± 0.56%) was on average also the smallest strain (12.4 ± 0.22 g) and body fat % was positively correlated with weight overall (Figure 3B, rho = 0.56, *p* <2.2 × 10^−16^), the largest average CC strain was not necessarily the most susceptible to developing obesity (Figures 2A,B, Supplementary Figure 3). For example, the CC strains with the highest average weight (23.4 ± 0.97 g in CC011/Unc, 22.4 ± 1.08 g in CC028/GeniUnc, and 22.3 ± 0.7 g in CC008/GeniUnc) did not necessarily always have the highest body fat % (15.7 ± 1.29% in CC011/Unc, 23.11 ± 1.59% in CC028/GeniUnc, and 15.00 ± 1.39% in CC008/GeniUnc).

### Adiposity Exhibits Complex Associations Across Various Health Measures at Baseline

Excessive adiposity is a risk factor for metabolic dysfunction and thus we quantified the relationship between circulating plasma analyte levels and body fat % (Figure 3). For example, for traits associated with metabolic syndrome such as total weight, circulating glucose, insulin, TG, and cholesterol, body fat % was significantly correlated with weight (rho = 0.56, *p* <2.2 × 10^−16^), insulin (rho = 0.44, *p* = 8.8 × 10^−11^), and TG (rho = 0.24, *p* = 5.9 × 10^−4^) as shown in Figures 3B–D, but not glucose nor cholesterol. In terms of metabolites associated with cardiovascular health, adiposity was not correlated with the risk factor TMAO but was moderately associated with circulating choline (rho = 0.190, *p*_*adj*_ = 0.012), carnitine (rho = 0.17, *p*_*adj*_ = 0.023), and phosphocholine (rho = 0.260, *p*_*adj*_ = 0.001; Figure 3A, Supplementary Table 5).

### Estimates for Broad Sense Heritability (H^2^) Show the Size of Strain Effects on Phenotypic Variation at Baseline

We next calculated broad sense heritability (H^2^) of traits at baseline to quantify the degree that genetic variation influences phenotypic variation compared to the variation of environmental factors. Linear regression analysis was performed to test whether strain had significant effects on phenotypic variation. Strain was a significant predictor for all traits at baseline except for circulating non-esterified fatty acids (Supplementary Table 4). Using the between- and within-strain mean square values (MSB and MSW, respectively) derived from these linear models, broad sense heritability (H^2^) was estimated by calculating the intraclass correlation (r_I_) and coefficient of genetic determination (g^2^) which determine the proportion of phenotypic variation accounted for by differences between strain (genetic variation) (Table 1). Estimates of H^2^ for phenotypic variation based on g^2^ were 0.359–0.565. The highest and lowest estimates of H^2^ were for lean mass (g^2^ = 0.565) and circulating non-esterified fatty acids (g^2^ = 0.029). Our assessment of H^2^ demonstrates that genotypic variation accounts for a large proportion of phenotypic variation in the CC for all body composition traits and a medium proportion of phenotypic variation for traits related to 1-carbon metabolism. We note that not all analytes were highly heritable.

**Table 1:** Broad sense heritability for baseline traits.

**Trait**	**Baseline r_**I**_**	**Baseline g^**2**^**
Body fat %	0.554	0.383
Lean %	0.529	0.359
Total weight	0.666	0.499
Fat mass	0.560	0.389
Lean mass	0.722	0.565
TG	0.622	0.452
Cholesterol	0.634	0.464
Glucose	0.259	0.149
Insulin	0.266	0.153
Glucose/Insulin	0.400	0.250
Albumin	0.515	0.347
ALT	0.407	0.255
AST	0.254	0.146
Creatinine	0.213	0.119
NE fatty acids	0.057	0.029
Urea/BUN	0.392	0.244
Betaine	0.621	0.450
Carnitine	0.398	0.249
Choline	0.329	0.197
Phosphocholine	0.419	0.265
TMAO	0.593	0.421
Metabolic health score	0.341	0.206

*Heritability estimates were calculated for traits at baseline using all mice. For each baseline trait, MSB and MSW values were derived from linear models with strain and week as covariates. Estimations of broad sense heritability were calculated for each trait represented by intraclass correlations (r_I_), which may be interpreted as the proportion of total phenotypic variation that is accounted for by differences between strains, and coefficients of genetic determination (g^2^), which accounts for the additive genetic variance that doubles during inbreeding. Since the CC is a recombinant inbred panel, g^2^ may be a more appropriate estimate for broad sense heritability in this study. However, other studies sometimes only provide one estimate of heritability or the other, so we present both values to facilitate comparisons with other findings in the literature*.

### Genetic Background Mediates Degree of Weight Gain, Adiposity, and Metabolic Health in Response to Diet

**Figure 4:**
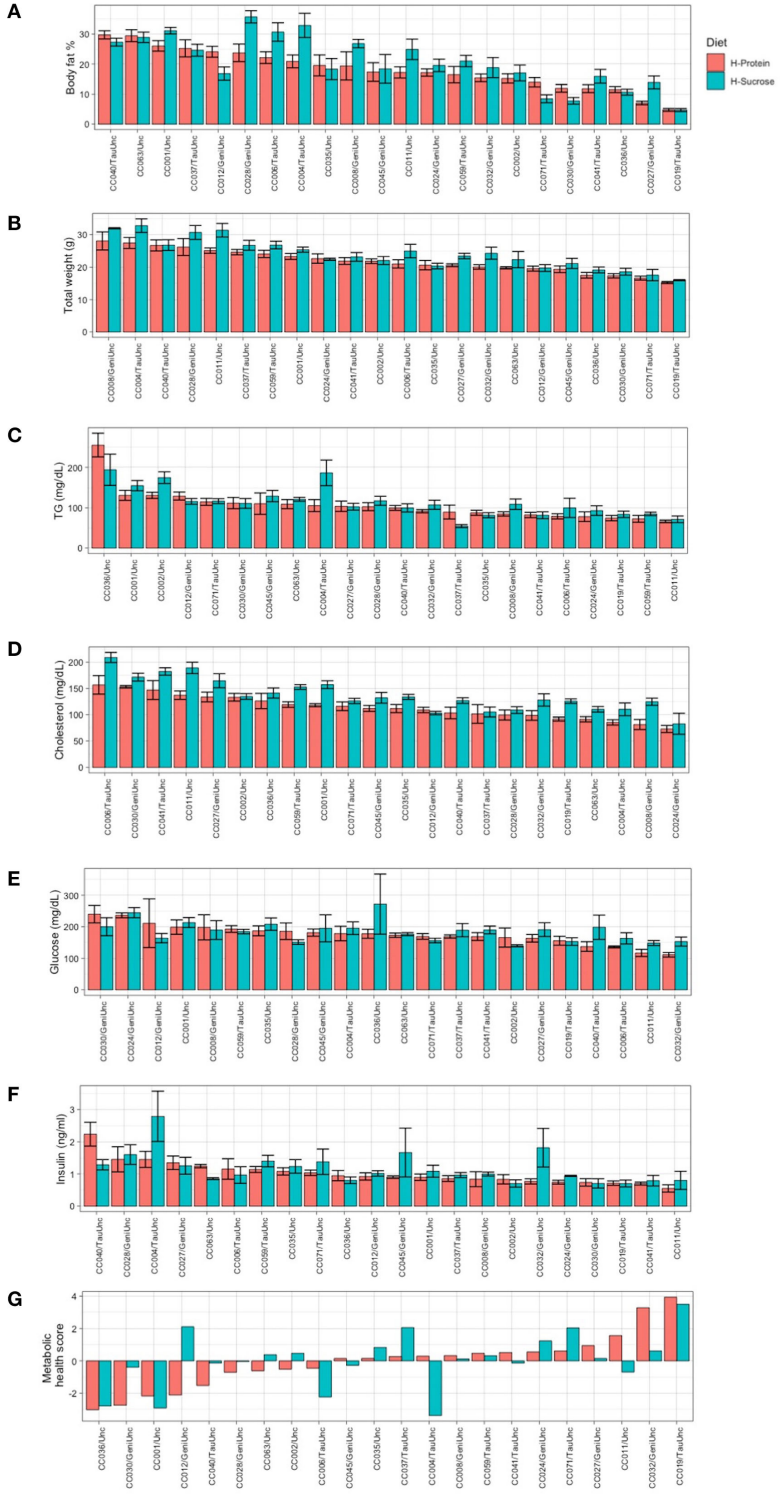
Average post-diet body fat %, total weight, circulating metabolic health marker levels, and metabolic health score by strain and diet show greater phenotypic variation by strain than diet. Phenotypic variation showed greater dependence on CC strain than experimental diet. Post-diet measurements of metabolic phenotypes are shown by diet for **(A)** body fat %, **(B)** total weight, **(C)** triglycerides (TG), **(D)** total cholesterol, **(E)** glucose, **(F)** insulin, **(G)** metabolic health score for each CC strain. CC strains in **(A–F)** are arranged in descending order based on HP diet. CC strains for **(G)** metabolic health score are ordered left to right from least healthy to most healthy. Data are mean ± SE for **(A–F)**; data are mean for **(G)**. For body fat % and weight, there were 4–5 mice per strain per diet except for CC024/GeniUnc (*n* = 2 per diet) and CC063/Unc (*n* = 3 per diet). For TG, cholesterol, glucose, insulin, and metabolic health score, 8–10 mice were available per strain, except for CC024/GeniUni (*n* = 2 per diet), CC063/Unc (*n* = 3 per diet), and CC071/TauUnc (HP *n* = 5, HS *n* = 3). H-Protein and H-Sucrose represent the HP and HS diets, respectively.

After establishing baseline phenotype values to examine the effect of strain without the influence of diet, we investigated the effect of diet in the CC population on weight gain and metabolic health. To accomplish this, we randomized the 204 female mice from 22 CC strains to one of two diets and challenged them for 8 weeks with either a high protein (*n* = 102) or high fat high sucrose diet (*n* = 102). After 8 weeks on the experimental diets, we assessed whether phenotypic response to diet differed by genetic background (CC strain) (see Methods and Figure 1). MRI body composition analysis of the CC mice after 8 weeks on the diet challenge revealed that diet influenced susceptibility to adiposity in a strain-dependent manner (Figure 4A). Strain CC028/GeniUnc was most susceptible to increased adiposity on the high fat high sucrose (HS) diet (35.7 ± 2.0%) and strain CC019/TauUnc was least susceptible (4.68 ± 0.5%) (Figure 4A, Supplementary Figure 4). CC040/TauUnc was most susceptible to increased adiposity on the high protein (HP) diet (29.7 ± 1.37%) and strain CC019/TauUnc was least susceptible (4.7 ± 0.47%). The effect of diet was highly variable across the selected strains from the CC. For example, CC028/GeniUnc and CC004/TauUnc had a 12% increase in adiposity when fed the HS diet compared to the HP diet (Supplementary Figure 4), while CC019/TauUnc and CC063/Unc showed negligible differences in adiposity when fed different diets (0.05 and 0.54%). Comparisons of phenotypic differences between baseline and post-diet body fat % (Supplementary Figure 5A) by strain and diet further emphasize the strain-dependent response of body fat % to diet in the CC.

Similar to adiposity, total weight, circulating analyte levels, and metabolic health score all showed phenotypic variation and different responses to diet depending on CC strain (Figure 4), though to a lesser degree than adiposity. As shown in Figure 4G, strain effects account for the inherent phenotypic variation in metabolic health illustrated by metabolic health score, as well as the varied responses to diet. Certain strains such as CC059/TauUnc and CC008/GeniUnc showed very little responses to diet in terms of their metabolic health score, while other strains showed improved metabolic health on either the HP compared to the HS diet (CC032/GeniUnc and CC004/TauUnc) or HS compared to the HP diet (CC012/GeniUnc and CC030/GeniUnc).

To ascertain whether there is a significant effect of CC strain x experimental diet interaction on adiposity and related traits, linear mixed models were fitted as appropriate for each trait using CC strain, experimental diet, and CC strain × experimental diet as covariates, followed by application of the Satterthwaite approximations for degrees of freedom for evaluating significance (Supplementary Table 6). A significant effect of CC strain × diet interactions was found for adiposity, fat mass, lean mass percentage, metabolic health score, and circulating TMAO and TG. The models for each phenotype were significant with the range of significant *p*-values from *p* = 7.37 × 10^−5^ for adiposity to *p* = 0.03 for metabolic health score (*F* = 3.36 and *F* = 1.84). There was a significant effect of strain on circulating cholesterol, glucose, and insulin (Supplementary Table 6), but no significant effect of CC strain × diet interactions which suggests that genotypic variation is largely responsible for the phenotypic variation of these traits.

To determine the magnitude of the effect of diet behind gene × environmental effects found for each trait in our linear mixed models, we performed Spearman's correlation analysis between the F-statistic of the gene x diet interactions of our models and Hedges' effect size for both diets (|g|), which demonstrated that the significance of gene x diet interactions were not significantly affected by diet for either diet (HP *p* = 0.96, HS *p* = 0.74). Furthermore, we categorized each trait as either largely affected by diet (|g| > 0.8) or not (|g| <0.8), and significantly affected by gene × diet interactions (*p* <0.05) or not (*p* > 0.05), followed by Chi square analysis of whether the effect size of diet and the gene x environment are significantly related. The results of the Chi square analysis (*p* > 0.99) were consistent with the results of the Spearman's correlations performed between the F-statistic of the gene x diet interactions models and Hedges' effect size for both diets (|g|) which suggest that the magnitude of the effect of diet is not a significant “driver” of gene x diet interactions.

### Magnitude of Quantified Diet Effects Varies Depending on Diet Macronutrient Composition for Body Composition and Obesity-Related Traits

Because diet is an important environmental factor that affects the manifestation of phenotypes, we next investigated the relative effect size of diet on clinical traits associated with adiposity and metabolic health. To accurately quantify the effect size of diet on each phenotype, Hedges' g was calculated for each trait instead of Cohen's d because strain groups were dissimilar in sample size for various traits. The difference in *n* by strain may result in unequal measures of variation between experimental groups, which needs to be adjusted for so that the standard deviation (SD) used to calculate effect size more closely reflects the SD of the population. Hedges' g uses pooled SD weighted by sample size of each group in its calculation (see Methods), which makes it a more appropriate measure of effect size when experimental groups are dissimilar in sample size compared to Cohen's d (Ellis, 2009). As shown in Table 2, the HS diet had large effects on circulating choline, urea and non-esterified fatty acids (NEFAS), as well as most traits associated with body composition (|g|> 0.8); medium effects on adiposity, glucose/insulin ratio, TMAO, and albumin (0.8 > |g| > 0.5); and small to negligible on all other phenotypes (|g| <0.5). In contrast, the HP diet only had large effects on total weight (|g|> 0.8); medium effects on glucose/insulin ratio, circulating choline, TMAO, NEFAS, albumin, urea, cholesterol, and TG, as well as all phenotypes associated with body composition; and small to negligible on all other phenotypes (|g| <0.5).

**Table 2:** Heritability estimations and Hedges' g diet effect sizes for post-diet traits.

**Trait**	**Post-diet r_**I**_ (full)**	**Post-diet r_**I**_ (partial)**	**HP r_**I**_**	**HS r_**I**_**	**Post-diet g^**2**^ (full)**	**Post-diet g^**2**^ (partial)**	**HP g^**2**^**	**HS g^**2**^**	**HP hedges' g**	**HS hedges' g**	**ICC of diet**
Body fat %	0.626	0.613	0.679	0.710	0.456	0.442	0.514	0.551	0.510	0.754	0.085
Lean %	0.619	0.598	0.679	0.671	0.449	0.426	0.514	0.505	−0.557	−0.810	0.133
Total weight	0.670	0.631	0.672	0.681	0.503	0.461	0.506	0.517	0.834	1.136	0.229
Fat mass	0.677	0.659	0.694	0.669	0.512	0.491	0.531	0.502	0.662	0.940	0.121
Lean mass	0.729	0.709	0.744	0.704	0.573	0.549	0.592	0.543	0.714	0.969	0.143
TG	0.547	0.543	0.464	0.639	0.377	0.372	0.302	0.469	−0.618	−0.423	0.027
Cholesterol	0.587	0.502	0.504	0.704	0.416	0.335	0.337	0.544	−0.798	−0.124	0.416
Glucose	0.244	0.239	0.351	0.125	0.139	0.136	0.213	0.067	0.318	0.426	0.033
Insulin	0.291	0.324	0.447	0.204	0.170	0.193	0.287	0.113	−0.439	−0.393	0.006
Glucose/Insulin	0.303	0.304	0.433	0.202	0.178	0.179	0.276	0.112	0.640	0.688	−0.010
Albumin	0.361	0.361	0.271	0.495	0.220	0.220	0.157	0.329	−0.712	−0.619	−0.002
ALT	0.222	0.223	0.281	0.248	0.125	0.125	0.164	0.141	−0.268	−0.337	−0.005
AST	0.219	0.216	0.234	0.180	0.123	0.121	0.132	0.099	−0.210	−0.326	0.018
Creatinine	0.175	0.172	0.174	0.114	0.096	0.094	0.095	0.061	−0.497	−0.364	0.025
NE Fatty acids	0.137	0.135	0.043	0.198	0.073	0.072	0.022	0.110	−0.794	−0.967	0.016
Urea/BUN	0.535	0.348	0.490	0.533	0.366	0.210	0.324	0.364	0.546	−0.800	0.610
Betaine	0.470	0.390	0.530	0.427	0.307	0.242	0.361	0.272	−0.331	0.473	0.366
Carnitine	0.424	0.411	0.491	0.321	0.269	0.258	0.326	0.191	−0.035	0.263	0.081
Choline	0.258	0.256	0.123	0.319	0.148	0.147	0.066	0.190	−0.781	−0.841	0.016
Phosphocholine	0.270	0.264	0.215	0.190	0.156	0.152	0.120	0.105	−0.417	−0.258	0.045
TMAO	0.363	0.344	0.436	0.332	0.221	0.208	0.279	0.199	−0.674	−0.622	0.107
Metabolic health score	0.328	0.329	0.390	0.391	0.196	0.197	0.242	0.243	−0.041	0.026	−0.010

*Post-diet heritability estimates were calculated from linear models including strain, diet, and week as covariates [Post-diet (Full)] and from linear models that only included strain and week as covariates [Post-diet (Partial)]. Diet-specific estimations of broad sense heritability were calculated for each trait represented by intraclass correlations (r_I_) and coefficients of genetic determination (g^2^) for each trait using the MSB and MSW for strain derived from linear models with strain and week as covariates using only data from each experimental diet per model as indicated to assess how different diet “environments” affect heritability. Hedges' g diet effect size values for HP and HS diets as compared to the baseline diet on post-diet traits were calculated to estimate the magnitude of effect size for each diet, with the sign indicating the direction of change between diets. Positive Hedges' g indicates increased phenotype values post-diet compared to baseline, e.g., body fat % was increased from baseline in mice after feeding them the HP diet. The intraclass correlation (ICC) for diet, which is the proportion of the total phenotypic variation that is accounted for by differences between diet, was calculated to compare the proportion of phenotypic variation attributed to diet in general or genetics*.

**Figure 5:**
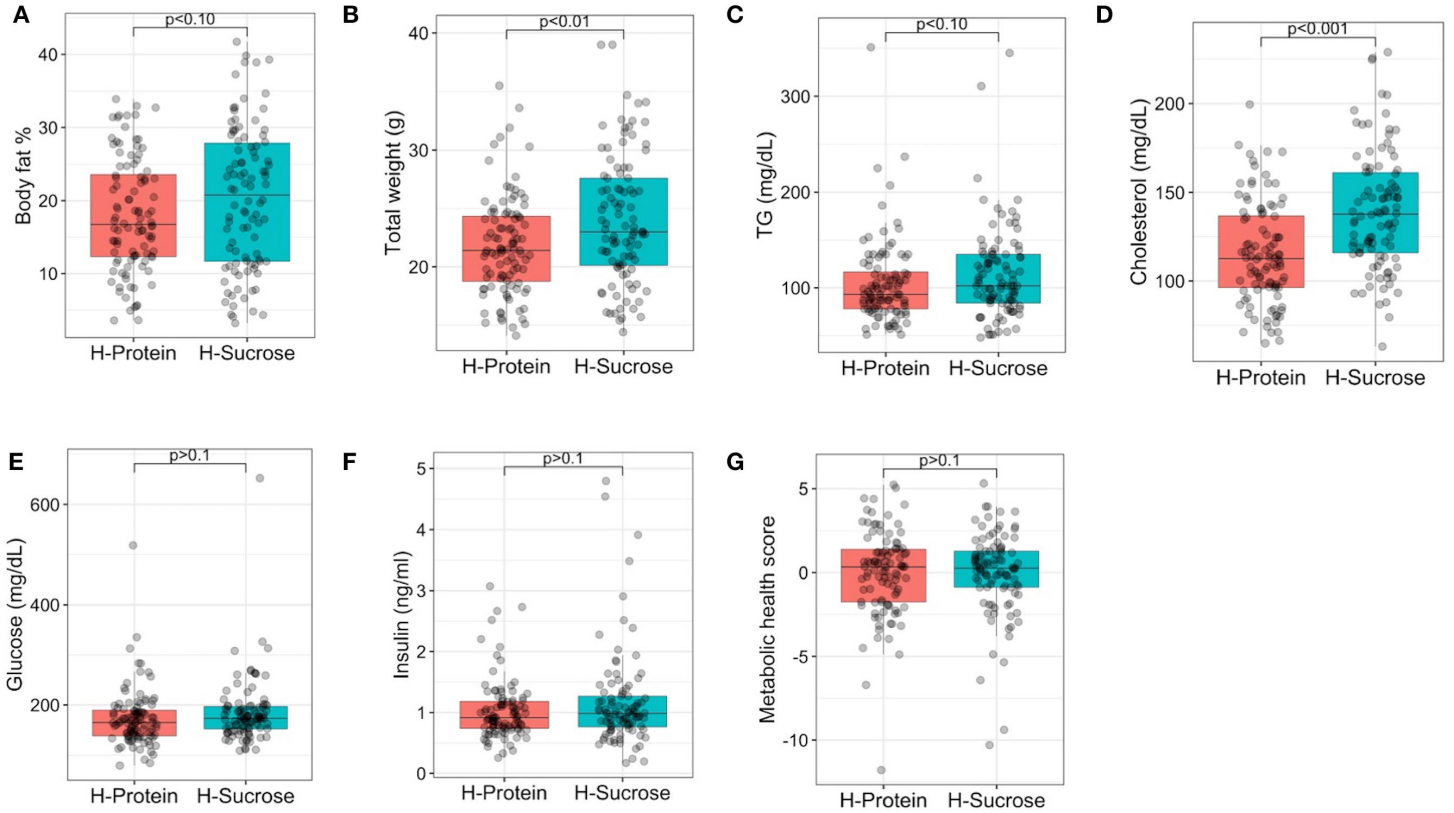
Average post-diet body fat %, total weight, circulating metabolic health marker levels, and metabolic health score by diet. Post-diet measurements of phenotypes are shown for **(A)** body fat %, **(B)** total weight, **(C)** triglycerides (TG), **(D)** total cholesterol, **(E)** glucose, **(F)** insulin, and **(G)** metabolic health score by diet after 8 weeks of feeding the experimental diets as indicated. Points are measurements obtained for each mouse. Linear mixed model analysis revealed that experimental diet alone did not have a significant effect in general on body fat %, TG, glucose, insulin, nor metabolic health score, but experimental diet did have significant effects on total weight (*p* < 0.01) and cholesterol (*p* < 0.001). For body fat % and weight, there were 4–5 mice per strain per diet except for CC024/GeniUnc (*n* = 2 per diet) and CC063/Unc (*n* = 3 per diet). For TG, cholesterol, glucose, insulin, and metabolic health score, 8–10 mice were available per strain, except for CC024/GeniUni (*n* = 2 per diet), CC063/Unc (*n* = 3 per diet), and CC071/TauUnc (HP *n* = 5, HS *n* = 3). H-Protein and H-Sucrose represent the HP and HS diets, respectively.

Post-diet values for body fat %, clinical blood chemistries, and circulating metabolites were established for each diet to assess the degree of phenotypic variation due to differences in macronutrient composition of diet (Figure 5). For both diets, there was a wide range of phenotypic variation within each diet for body fat % (HP = 3.6–33.9%, HS = 3.17–41.7%), total weight (HP = 14.1–35.5 g, HS = 14.1–39 g) and cholesterol (HP = 64.8–199.5 mg/dL, HS = 63–228.9 mg/dL). Means per diet for body fat % (HP = 17.95 ± 0.77%, HS = 20.31 ± 0.97%), total body weight (HP = 21.78 ± 0.42 g, HS = 24.02 ± 0.5 g), TG (HP = 104.6 ± 4.28 mg/dL, HS = 113.65 ± 4.73 mg/dL), cholesterol (HP = 115.1 ± 2.85 mg/dL, HS = 139.51 ± 3.3 mg/dL), glucose (HP = 173.45 ± 5.73 mg/dL, HS = 184.63 ± 6.42 mg/dL), and insulin (1.03 ± 0.05 ng/ml, HS = 1.17 ± 0.08 ng/ml) showed slightly elevated values for each trait on the HS diet compared to the HP diet (Supplementary Table 7), but the only significant increases in phenotype were for total weight and cholesterol (*p* <0.01, Student's t-test), not body fat %, TG, glucose, insulin, nor metabolic health score (Figure 5). Relative to mice fed the HP diet, mice fed the HS diet showed a 10.6% increase in total weight (Figure 5B) and a 21.2% increase in cholesterol (Figure 5D), suggesting that macronutrient composition had a stronger effect on these traits compared to body fat %, TG, glucose, insulin, and metabolic health score.

To further assess whether diet had a significant effect on adiposity and related phenotypes, linear mixed model analysis was performed (Supplementary Table 8), which showed that experimental diet had a significant effect on all phenotypes related to body composition, except post-diet body fat % for which diet showed a suggestive effect (*F* = 3.98, *p* = 0.057). Although experimental diet alone did *not* have a significant effect in general on body fat %, TG, glucose, insulin, nor metabolic health score (Figure 5), experimental diet *did* have significant effects on total weight (*F* = 20.0, *p* = 0.0002) and cholesterol (*F* = 43.8, *p* = 6.22 × 10^−7^). Furthermore, experimental diet also had significant effects on circulating urea, betaine, TMAO, carnitine, and phosphocholine (Supplementary Table 8), indicating that diet macronutrient composition still plays an important role in terms of metabolic health.

To confirm the degree to which genetic background mediates weight gain, adiposity, and metabolic health in response to diet, additional linear mixed model analyses with strain, diet, and strain × diet interactions as all random effects were performed for each trait to estimate the relative heritable variation that can be attributed to genetics, environment (diet), and gene × environmental effects. From the results of these models, we calculated the variance for each of these terms (Supplementary Table 9) and found that a large proportion of relative phenotypic variation can be attributed to background strain for most traits, especially body fat %, total weight, and TG (>~49.6%). In contrast, the proportion of relative phenotypic variation that can be attributed to diet varied depending on the trait, where cholesterol, betaine, and urea/BUN were the traits that had the highest proportion of heritable variation attributed to diet (>~21%).

### Post-diet Estimates for Broad Sense Heritability (H^2^) Reaffirm the Strong Contribution of Strain Effects on Heritable Phenotypic Variation and Identify Traits With High Proportions of Heritable Phenotypic Variation Attributed to Diet

The degree to which genetics, diet, and gene x diet interactions influence phenotypic variation differs depending on the trait. To quantify the relative heritable phenotypic variation which can be attributed to genetics or diet for body fat % and obesity-related traits, we calculated heritability using the mean square between (MSB) strains and mean square within (MSW) strains derived from two different linear models for post-diet traits (a “full” additive model that includes strain, diet, and week as variables and a “partial” model that excludes diet) and the intraclass correlation (ICC) for diet using the mean square between (MSB) diets and mean square within (MSW) diets derived from the “full” model (Methods). Heritability estimates were similar for most traits regardless of the model used (“full” vs. “partial”) except for traits where the ICCs for diet were relatively high, such as total weight, cholesterol, urea/BUN, and betaine, demonstrating the robust contribution of strain to heritable variation compared to diet (Table 2). The relatively high diet ICCs for total weight, cholesterol, urea/BUN, and betaine suggest that diet may be responsible for a higher proportion of heritable variation for these traits compared to other traits, which is consistent with the results of our linear mixed models testing the significance of diet that also show diet as significantly affecting these traits (Supplementary Table 8). Traits with negative or close to zero diet ICCs had higher within-diet variation than between-diet variation. Interestingly, with the exception of insulin and metabolic health score, most post-diet traits had higher heritability estimates when the MSB term was used from linear models that included diet compared to the models excluding diet, suggesting that accounting for the effect of diet improved heritability estimates since either the within-strain variation was decreased and/or the between-strain variation was increased.

Diet-specific heritability was also calculated using linear models fitted only including mice fed HP or HS diet with strain and week as covariates to compare changes in heritability for each experimental diet due to “environmental” differences (Tables 1, 2). One caveat of comparing baseline heritability and diet-specific post-diet heritability is that diet-specific post-diet heritability values were calculated using half the number of mice as the baseline heritability values, which could affect the within-strain variance component of the heritability calculations. Nonetheless, assuming that the genotypic variance is the same between diets and time points (baseline vs. post-diet), we can still identify which traits may be more strongly affected by differences in macronutrient composition. Indeed, after calculating the heritability estimates for each of the traits post-diet on the respective experimental diets, we found that the different “environments” (diets) resulted in slight alterations in heritability estimates depending on the trait. For example, the difference in macronutrient composition appears to have a bigger impact on traits such as cholesterol, insulin, and glucose with larger variation in heritability (Table 2), and less important to traits such as metabolic health score where heritability estimates remain consistent (baseline g^2^ = 0.21, HP g^2^ = 0.24, HS g^2^ = 0.24).

### Comparison of Quantified Metabolic Traits During Daytime and Nighttime Show Decreased Rates of Metabolism, Energy Intake, Utilization of Carbohydrates as a Fuel Source, and Basal Activity During Rest

Obesity is characterized by the excess accumulation of body fat, which results from chronic energy imbalance between energy intake and expenditure. Given the diverse range of body fat accumulation in response to diet across strains, we sought to elucidate the differences in metabolism between strains on each diet by using indirect calorimetry to measure the following traits related to energy balance in mice after 8 weeks on the experimental diets: (1) heat expenditure to estimate metabolism levels, (2) respiratory exchange rate (RER) to estimate substrate utilization (carbohydrate compared to fat as a source of energy), (3) food intake to estimate energy consumption, and (4) basal activity. Energy consumption was calculated by multiplying feed consumption measurement (in grams) by the calorie (kcal) content per gram feed for each diet. Similar to other phenotypes reported above, linear mixed model analysis was performed for each trait to test whether experimental diet, CC strain, and/or CC strain x experimental diet interactions had significant effects on metabolic traits.

**Figure 6:**
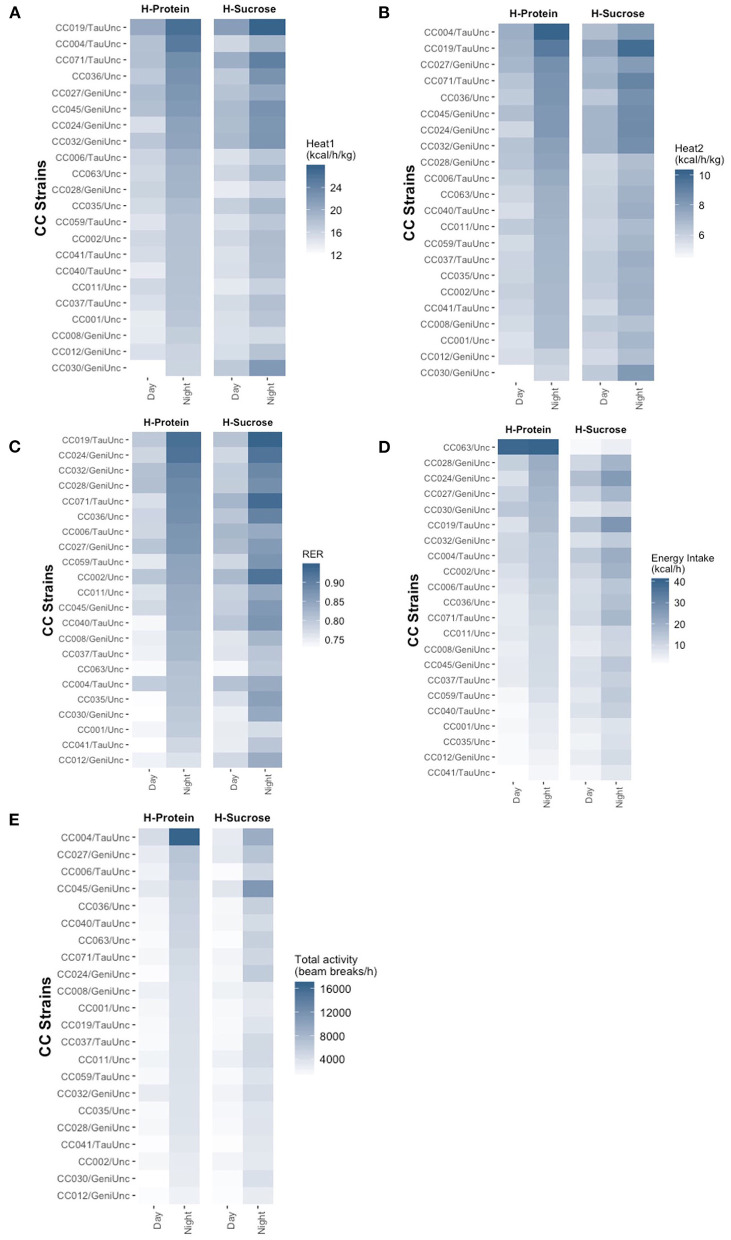
Dietary effects of heat expenditure, energy intake, RER, and activity in the Collaborative Cross. Similar to body composition, circulating analytes, and metabolic health score, phenotypic variation of metabolic traits showed greater dependence on CC strain than experimental diet. Post-diet quantification of average **(A)** heat expenditure adjusted for total body mass (kcal/h/kg), **(B)** heat expenditure adjusted for lean mass (kcal/h/kg), **(C)** RER, **(D)** energy intake (kcal/h), and **(E)** total basal activity (beam breaks/h) for each CC strain on each diet shows range of variation across strains for metabolic traits. Strains are ordered in descending order by HP diet. For metabolic traits, there were 4–5 mice per strain per diet except for CC024/GeniUnc (*n* = 2 per diet) and CC063/Unc (*n* = 3 per diet). H-Protein and H-Sucrose represent the HP and HS diets, respectively.

Heat production, RER, energy intake, and basal activity phenotypes varied widely by CC strain (Figure 6, Supplementary Figures 6, 7, Supplementary Table 10), with phenotype measurements higher at night than day which reflected the active nocturnal behavior of mice. Wilcoxon signed rank tests performed comparing the day and night measurements for each trait confirmed the differences between light and dark cycles for all strains on both diets (*p* <2.2 × 10^−16^ for all traits). Overall heat production while accounting for total weight (Heat1) was highest on average for both day and night cycles in the leanest strain, CC019/TauUnc on the HS diet (21.4 ± 0.64 and 27.6 ± 0.87 kcal/h/kg in the day and night) (Figure 6A, Supplementary Figure 6A). During the day, heat production was lowest in CC030/GeniUnc on the HP diet (11.9 ± 2.86 kcal/h/kg) even though CC030/GeniUnc was relatively lean compared to other strains on the same diet (11.9 ± 1.2%, Figure 4A), while heat production was lowest in CC008/GeniUnc during the night on the HS diet (15.4 ± 0.30 kcal/h/kg), which was one of the fatter strains compared to other strains in the same diet (26.8 ± 1.3%, Figure 4A). Overall heat production while accounting for *only lean mass* (Heat2) (Figure 6B, Supplementary Figure 6B) was highest on average for CC019/TauUnc on the HS diet during the day (7.61 ± 0.23 kcal/h/kg) and CC004/TauUnc on the HP diet at night (10.2 ± 0.56 kcal/h/kg) and lowest in CC030/GeniUnc on the HP diet (4.35 ± 1.06 and 5.81 ± 0.94 kcal/h/kg in the day and night, respectively). In summary, variation in energy production was much larger between strains than diets, with the differences in phenotype by diet depending on the strain (Supplementary Figure 6). Linear mixed model analysis showed that CC strain × experimental diet interactions had significant effects on energy production during both day and night, but the effect of CC strain was much stronger and may be driving the effects of CC strain × experimental diet interactions (Supplementary Table 6). In linear mixed models testing the effect of experimental diet alone, diet had a significant effect on Heat2 during the day (*F* = 5.3, *p* = 0.03) but not any of the other heat production measured (Supplementary Table 8), suggesting that diet may have a different effect on metabolism during the day for lean mass compared to non-lean mass.

Similar to energy production, the wide range of variation in substrate utilization (RER) depended on CC strain and were all higher during the night compared to the day for mice within the same strain on either diets (Figure 6C, Supplementary Figure 7A). RER levels were lowest in CC030/GeniUnc during the day and CC012/GeniUnc during the night on the high protein diet (0.725 ± 0.007 and 0.76 ± 0.01) even though the adiposity of CC012/GeniUnc was twice the adiposity of CC030/GeniUnc (24.1 ± 1.8% and 11.9 ± 1.2%, Figure 4A), while RER levels were highest in CC071 during the day and CC019/TauUnc during the night on the HS diet (0.825 ± 0.019 and 0.943 ± 0.013) despite vastly different levels of adiposity (13.9 ± 1.5% in CC071 and 4.7 ± 0.5% in CC019/TauUnc, Figure 4A). Surprisingly, linear mixed model analysis revealed that both CC strain and experimental diet independently had significant effects on RER for both day and night (Supplementary Tables 6, 8), but despite CC strain having a stronger effect than diet, the effects of CC strain × diet interactions were not significant.

Our indirect calorimetry assays were also able to calculate the energy intake and activity of the mice over the 48-h test. As expected, there were significant differences between night and day cycles in both of these behaviors, as confirmed by the results of Wilcoxon signed rank tests (*p* <2.2 × 10^−16^). Energy intake was lowest in the lean strain CC041/TauUnc on the HP diet for both day and night (0.579 ± 0.110 kcal/h and 2.68 ± 0.915 kcal/h) (Figure 6D, Supplementary Figure 7B). Food intake was highest for strain CC024/GeniUnc during the day (16.47 ± 3.661 kcal/h) and CC019/TauUnc during the night (26.64 ± 7.301 kcal/h) on the HS diet. The energy consumption was variable depending on the diet consumed. For example, in terms of mice on the HS diet, energy intake was highest in CC024/GeniUnc during the day (16.47 ± 3.66 kcal/h) and CC019/TauUnc at night (26.64 ± 7.30 kcal/h), and lowest in CC063/Unc during both day (2.32 ± 0.17 kcal/h) and night (4.59 ± 0.44 kcal/h). Additionally, energy intake for CC063/Unc was extremely variable on the HP diet during the day and night (39.45 ± 18.00 kcal/h and 40.42 ± 17.34 kcal/h). Because of this high variability, four types of linear mixed models were fitted for both day and night energy intake: (1) model testing for the effect of diet including CC063/Unc, (2) model testing for the effect of diet excluding CC063/Unc, (3) model testing for the effect of CC strain × diet including CC063/Unc, and (4) model testing for the effect of CC strain x diet excluding CC063/Unc. For energy intake both day and night, both experimental diet and CC strain had significant effects on energy intake regardless of whether CC063/Unc was included, but the CC strain × diet interaction did not significantly affect energy intake when CC063/Unc was excluded (Supplementary Table 11). Although we could not identify a specific error with the collection or calculation of the data for CC063/Unc, results for energy intake from CC063/Unc should be interpreted with caution.

Basal activity exhibited phenotypic variation depending on and between CC strains, but barely any difference by diet (Figure 6E, Supplementary Figure 7C). Diurnal basal activity was lowest in CC030/GeniUnc on the HP diet and CC041/TauUnc on the HS diet (988.8 ± 383.1 and 1,188.2 ± 260.6 beam breaks/h), and highest in CC004/TauUnc on the HP diet and CC045/GeniUnc on the HS diet (4,328.1 ± 985.7 and 3,322.5 ± 988.8 beam breaks/h), while nocturnal basal activity was lowest in CC012/GeniUnc on the both HP and HS diets (2,304.4 ± 124.7 and 2,792.7 ± 337.7 beam breaks/h), and highest in CC004/TauUnc on HP and CC045/GeniUnc on HS diets (16,742.5 ± 1,919.9 and 11,081.9 ± 6,070.3 beam breaks/h). Linear mixed model analysis confirmed that only CC strain had a significant effect on both diurnal and nocturnal basal activity; the effects of experimental diet and CC strain x experimental diet interactions were not significant (Supplementary Tables 6, 8).

### Complex Relationships Between Adiposity, Energy Intake, and Energy Expenditure Suggest an Important Role of Diet Substrate Utilization in Maintaining Energy Homeostasis

**Figure 7:**
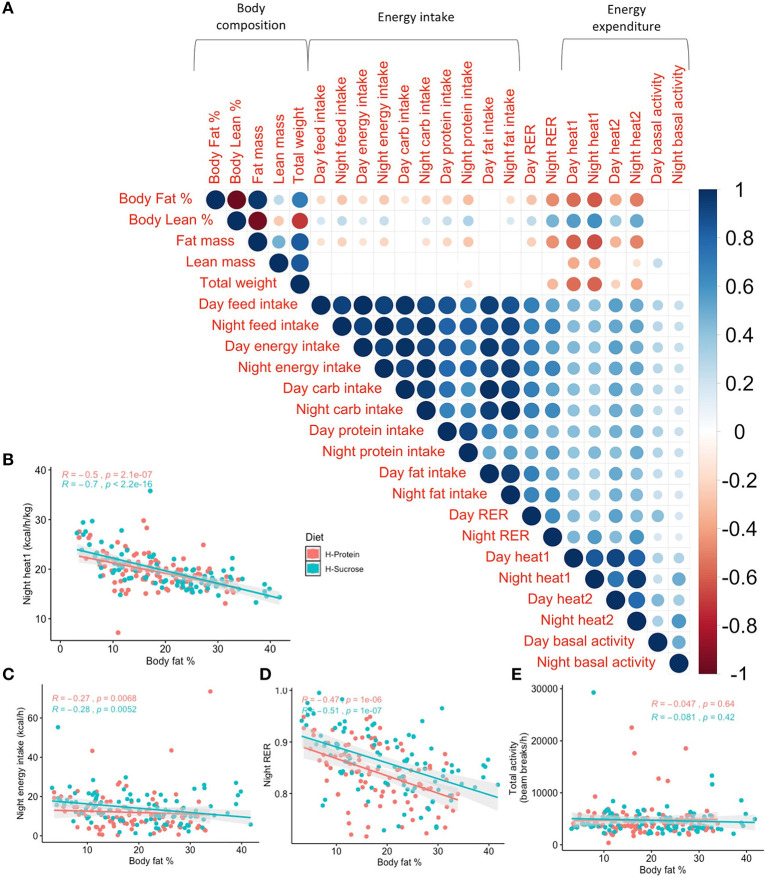
Post-diet Spearman's correlations of indirect calorimetry phenotypes contrast the difference in relationship between body fat % and each metabolic trait depending on time. Phenotypic correlations between body fat % and metabolic traits reveal stronger relationships between body fat % and energy expenditure than body fat % and energy intake regardless of the time of day. **(A)** Spearman's correlation of post-diet phenotypes assessed using indirect calorimetry with *p*-values adjusted using the Benjamini–Hochberg method. Only significant correlations (*P*_*adj*_ < 0.05) are shown. Scale indicates rho value. Spearman's correlations by diet between post-diet body fat % and nocturnal **(B)** heat production adjusted for total body weight (*R* < −0.49, *p* < 2.11 × 10^−7^), **(C)** energy intake (*R* < −0.269, *p* < 6.81 × 10^−3^), **(D)** RER (*R* < −0.471, *p* < 1.01 × 10^−6^), and **(E)** total basal activity (*R* < −0.0471, *p* > 0.419). *R* is Spearman's rho. H-Protein and H-Sucrose represent the HP and HS diets, respectively.

Our comprehensive phenotyping demonstrates the high variability among metabolic traits. Using the phenotyping data, Spearman's correlations between body composition and traits related to energy intake or expenditure were performed. Although the individual phenotypes are variable between strains there are several notable results, such as the negative correlations between body fat percentage and all expenditure phenotypes except for basal activity and diurnal fat intake (rho < −0.17, *p*_*adj*_ <0.02). Conversely, lean mass percentage was positively correlated with all energy intake/expenditure phenotypes except for basal activity, diurnal fat intake, and diurnal carbohydrate intake (rho > 0.16, *p*_*adj*_ <0.025, Figure 7). Total body weight was significantly but negatively correlated with nocturnal protein intake, night RER, and heat expenditure (rho < −0.17, *p*_*adj*_ <0.01), while all energy intake phenotypes were positively correlated with RER, energy expenditure phenotypes, and basal activity (rho > 0.19, *p*_*adj*_ <0.008, Figure 7). Body fat percentage and heat production (accounting for total weight) are negatively correlated for both day and night (rho = −0.563 and rho = −0.612), stronger than the negative correlations between body fat percentage and energy intake (rho = −0.20 and rho = −0.26). These data demonstrate that the decrease in food intake as body fat percentage increases is not enough to maintain energy balance.

**Figure 8:**
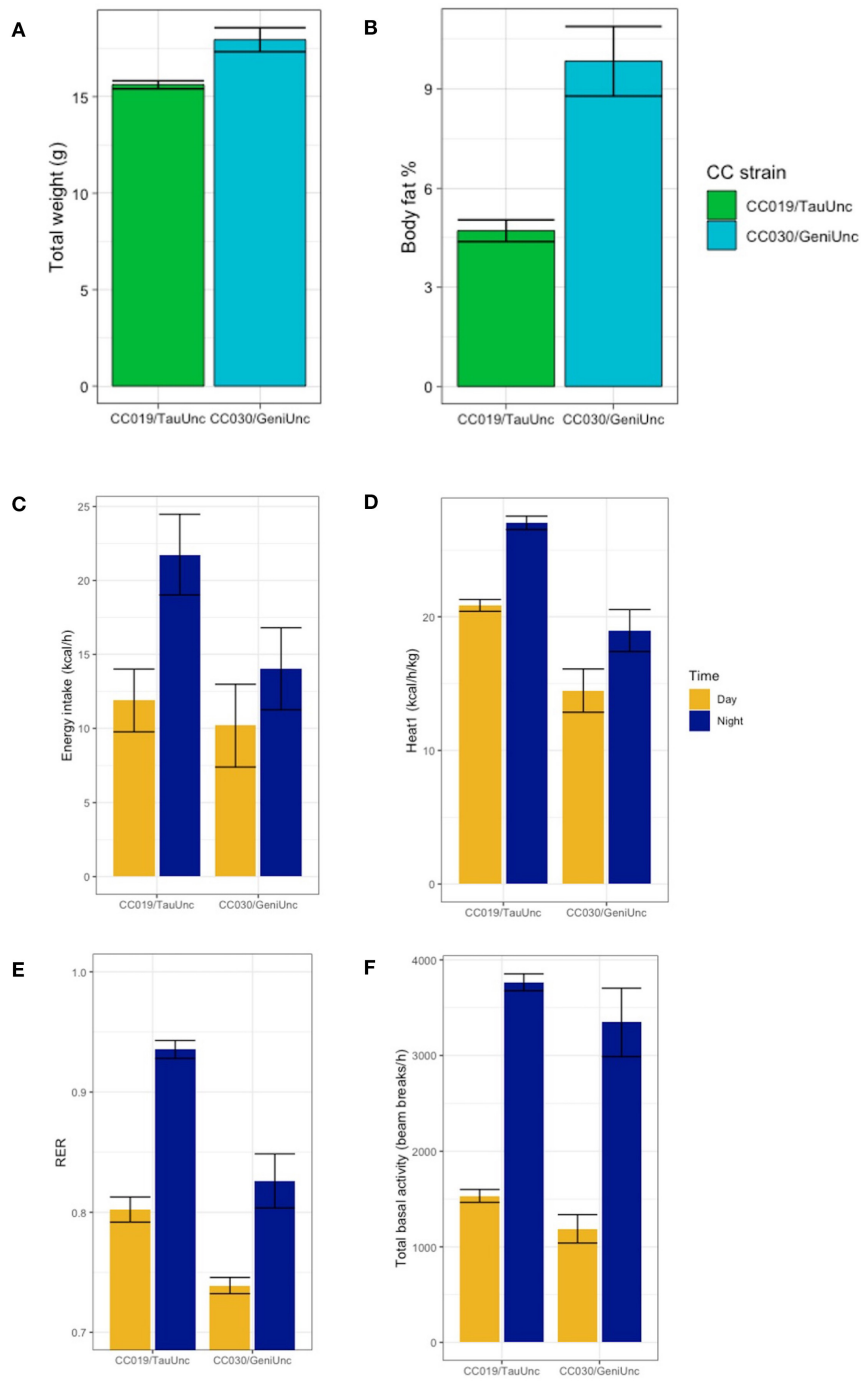
Strain-specific effects of diet on body fat %, heat expenditure, energy intake, activity, and RER. Examination of body composition and metabolic traits of two specific CC strains suggests different methods of maintaining energy balance for each strain. Post-diet quantification of average **(A)** total weight, **(B)** body fat %, **(C)** energy intake (kcal/h), **(D)** heat expenditure adjusted for total body mass (kcal/h/kg), **(E)** RER, and **(F)** total basal activity (beam breaks/h) for strains CC019/TauUnc and CC030/GeniUnc. Data are mean ± SE calculated using data from both diets for each strain.

Coupled with average adiposity measurements, indirect calorimetry data demonstrated that energy expenditure varies tremendously between inbred strains of similar weight, specifically strains CC030/GeniUnc and CC019/TauUnc (Figure 8). Although mice from these two strains were close in terms of average total body weight (Figure 8A), the average body fat percentage of CC030/GeniUnc was more than twice the average body fat percent of CC019/TauUnc (Figure 8B). CC019/TauUnc stayed consistently lean across diets, while CC030/GeniUnc's highest average post-diet body fat percentage paradoxically decreased with increasing dietary fat content (Figure 4A). Comparing the two strains of mice on the same diet, CC019/TauUnc mice consistently consumed more calories than CC030/GeniUnc mice during both day and night (Figure 8C) but also consistently produced more heat than CC030/GeniUnc and importantly, produced enough heat to achieve energy balance (Figure 8D). In addition to CC019/TauUnc's relatively high metabolism, the difference in substrate utilization between the two strains could help to explain their different responses to diet (Figure 8E); during the night, the average RERs of CC019/TauUnc are 0.943 and 0.926 on the HS and HP diets, and the average RERs of CC030/GeniUnc are 0.82 and 0.798 on the HS and HP diets, implying that CC019/TauUnc mice are utilizing carbohydrates as their fuel source more than CC030/GeniUnc mice, which could suggest that CC019/TauUnc mice are more active than CC030/GeniUnc mice. Intriguingly, substrate utilization during the light phase was quite different between strains. The average RERs of CC019/TauUnc across diets is 0.800 and the average RERs of CC030/GeniUnc are 0.746 and 0.725 on the HS and HP diets, which suggests that at rest CC030/GeniUnc mice preferentially utilize fat as an energy source more than carbohydrate as compared to CC019/TauUnc mice (Figure 8F).

### Small but Significant Alterations in Metabolite Levels Are Associated With Diet-Driven Adiposity, but Largely Not Associated With Metabolic Phenotypes

**Figure 9:**
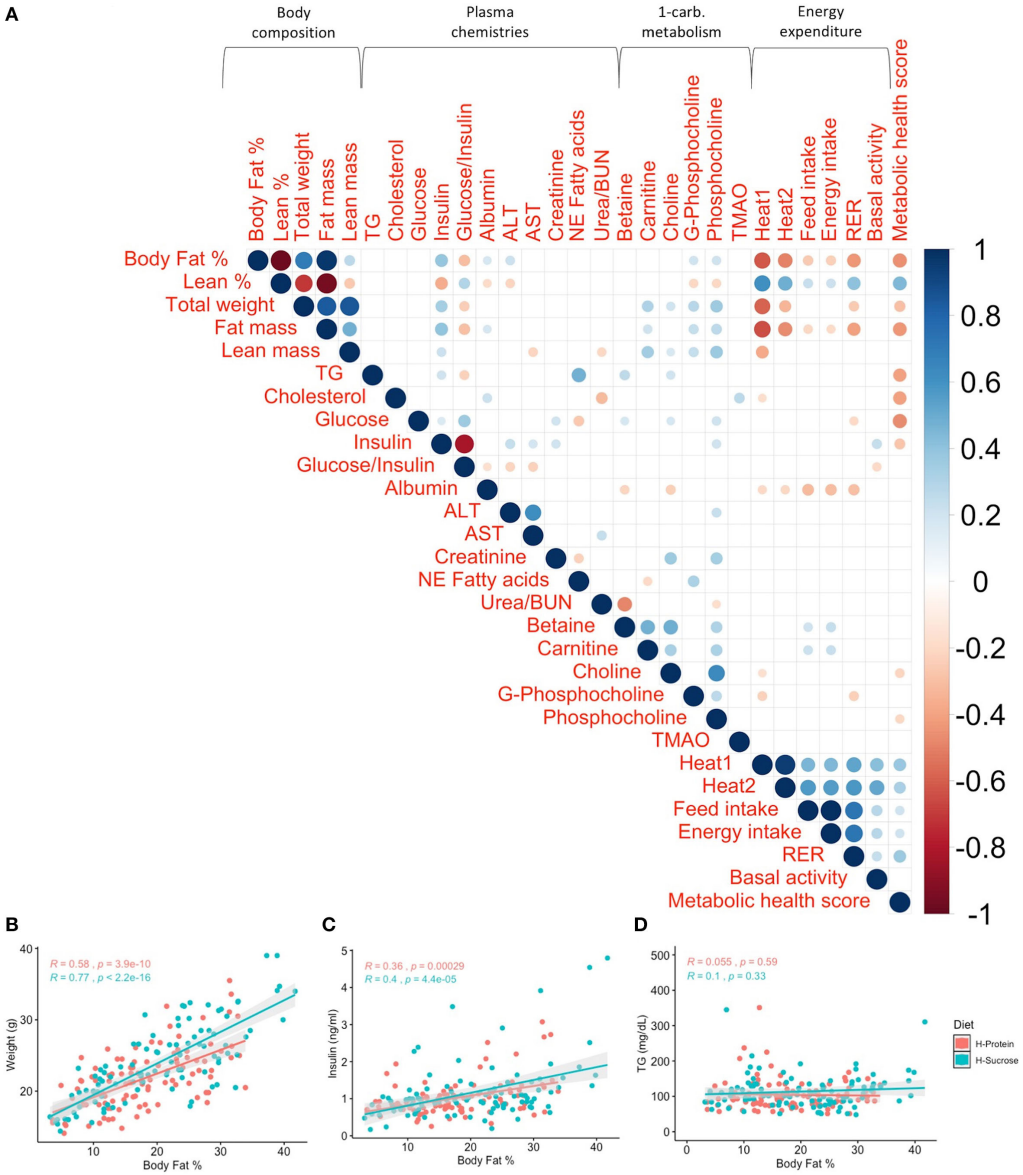
Post-diet phenotype correlations demonstrate that most relationships between traits are maintained after the diet challenge. Relationships between body fat % and weight, insulin, and metabolic health score are still strong after the diet challenge for both diets, while the association between body fat % and triglycerides (TG) is no longer significant. **(A)** Spearman's correlations of post-diet phenotypes with *p*-values adjusted using the Benjamini–Hochberg method. Only significant correlations (*p*_*adj*_ < 0.05) are shown. Scale indicates rho value. Spearman's correlations by diet between post-diet body fat % and **(B)** weight (*R* > 0.579, *p* < 3.91 × 10^−10^), **(C)** insulin (*R* > 0.359, *p* < 2.91 × 10^−4^), and **(D)** TG (*R* < 0.101, *p* > 0.329) show significant correlations between body fat % and weight, as well as body fat % and insulin, but not TG. R is Spearman's rho. H-Protein and H-Sucrose represent the HP and HS diets, respectively.

Given the variation in diet-driven changes in adiposity, we next investigated whether there were alterations in metabolic health in corresponding fashion. We correlated body fat % after diet feeding with other traits (Figure 9). Broadly, body fat % is strongly correlated with total weight (Figure 9B; rho > 0.579, *p* <3.91 × 10^−10^), and moderately correlated with insulin levels, total heat production, and total RER (Supplementary Table 12). Remarkably, the significant correlations at baseline (Figure 3) between body fat % and TG (rho = 0.24, *p*_*adj*_ = 1.35 × 10^−3^), carnitine (rho = 0.17, *p*_*adj*_ = 0.036), and choline (rho = 0.19, *p*_*adj*_ = 0.018) were no longer significant after the diet challenge (Supplementary Table 12), indicating that the metabolic effect of diet varies among clinical traits.

Spearman's correlation analysis performed between metabolic traits and other traits related to adiposity (Figure 9A) revealed significant correlations between metabolic health score and heat production accounting for total weight (Heat1) (rho = 0.37, *p*_*adj*_ = 2.61 × 10^−7^), heat production accounting for lean mass (Heat2) (rho = 0.32, *p*_*adj*_ = 9.12 × 10^−6^), feed intake (rho = 0.20, *p*_*adj*_ =0.009), energy intake (rho = 0.20, *p*_*adj*_ = 0.009), and RER (rho = 0.36, *p*_*adj*_ = 7.08 × 10^−7^) but not basal activity (*p*_*adj*_ = 0.76). These relationships are heavily driven by the body fat % component of metabolic health score, as body fat % was also significantly correlated with these traits but in the “opposite” direction (Supplementary Table 12). Of all circulating analytes and 1-carbon metabolites, RER was moderately correlated with albumin (rho = −0.29, *p*_*adj*_ = 3.21 × 10^−4^), heat production accounting for total body weight and lean mass showed a slight negative correlation with albumin (rho = −0.19, *p*_*adj*_ = 0.01 for both heat production estimations), and feed intake was positively correlated with betaine (rho = 0.21, *p*_*adj*_ = 0.02) and carnitine (rho = 0.22, *p*_*adj*_ = 9.12 × 10^−3^). Other than metabolic traits, the only traits which total basal activity showed slight correlations with were insulin (rho = 0.23, *p*_*adj*_ = 3.31 × 10^−3^) and glucose/insulin ratio (rho = −0.19, *p*_*adj*_ = 0.02).

## Discussion

With the rapid rise in the global prevalence of obesity and obesity-related diseases in the recent decades (Flegal, 2010; Ogden et al., 2016, 2018), there is a crucial need to improve our understanding of obesity. Individually, diet and genetics are known to be critical factors in the development of obesity, but our understanding of how diet and genetics interact to affect obesity remain to be fully elucidated. Taken at the individual gene level, this is a daunting task. There are hundreds of genes associated with body weight and BMI reported in the GWAS catalog and these can interact with each other and diet, increasing the complexity of obesity (Bell et al., 2005; Rankinen et al., 2006; Kunej et al., 2013; Singh et al., 2017). Thus, the complexity and heterogeneity of obesity may affect dietary recommendations, as illustrated by the lack of a universally “perfect” diet for weight loss (Dansinger et al., 2005; Johnston et al., 2014). Increasing our knowledge of how genetics and environmental factors (particularly diet) interact, the degree to which these interactions impact the development of obesity, and the mechanisms behind these effects are crucial to developing successful methods for mitigating obesity.

To investigate the degree that genetics, diet, and gene-by-diet interactions impact phenotypic variation in obesity, obesity-related traits, and metabolic traits, we performed our study on multiple strains of mice from the CC genetic reference population to overcome the limitations of human studies, especially in terms of controlling genetic background, diet, and other environmental influences. The CC is well-suited for investigating genetic vs. environmental impacts on phenotypic variation due to its high genetic diversity and ability to generate genetic “replicates” which enables increased accuracy in phenotypic measurements. Indeed, the CC has already been used to provide a genetic framework to depict the relationship between body weight and the central nervous system (Mao et al., 2015), high fat diet and fasting glucose levels (Atamni et al., 2016), and hepatic gene expression in response to impaired glucose tolerance (Atamni et al., 2019). The CC has also been used as a model to study exercise-induced paradoxical fat response (McMullan et al., 2018). The current manuscript adds to this literature by examining the dietary responses of the CC.

While previous studies have examined subsets of obesity-related traits in the CC and energy balance traits have been examined in pre-CC lines (Mathes et al., 2011), we examined the unique effect of diet in this population. In this study we sought to elucidate the relationships between genetic background, diet, adiposity, and obesity-related traits. Our comprehensive phenotyping included: body composition, circulating analyte and metabolite levels, and metabolism through indirect calorimetry, followed by the integration of all these data in common analyses. We found that in the absence of dietary perturbation, many of the traits phenotyped in this manuscript are heritable in the CC. Defined as the proportion of phenotypic variation due to genetic variation for a specific population, we calculated broad sense heritability for adiposity and other traits for mice on the synthetic chow diet at baseline to estimate the strength of genetic contribution. Traits related to body composition had moderately high broad sense heritability (g^2^) at baseline ranging between 0.359 and 0.565, with the broad sense heritability estimate of total body weight at 0.499 which is higher than a previously reported estimate in the CC at 0.37 (Atamni et al., 2016). Given that H^2^ estimates can vary among studies, we also calculated g^2^ for baseline body fat %, lean %, and total weight using four publicly available body composition data sets (McMullan et al., 2018). The range of g^2^ for these traits across the 4 data sets were between 0.268 and 0.511, similar to the estimates in this study. The average baseline g^2^ for lean % in the McMullan study (g^2^ = 0.358) and the current study (g^2^ = 0.359) were closer than the average g^2^ for weight in the McMullan study (g^2^ = 0.357) and the current study (g^2^ = 0.499), but the average g^2^ for body fat % in the McMullan study and the current study was the same (g^2^ = 0.383), which is close to the minimum heritability of 0.4 in humans as indicated by twin studies (Bell et al., 2005). The heritability of most circulating metabolites in the CC varied between 12 and 46%, similar to the heritability of circulating small metabolites and amino acids in humans, which has been reported to vary between 23 and 55% (Dharuri et al., 2014). Interestingly, broad sense heritability for circulating insulin (0.153) was much lower than heritability for adiposity (0.383), which implies that environmental factors such as diet or lifestyle may have a stronger effect on attenuating hyperinsulinemia than adiposity. Overall, these data suggest similar overall metabolic health parameters to those observed in humans, demonstrating that the high genetic, and phenotypic diversity of the CC make this mouse panel a suitable model for studying obesity, a trait with complex etiology. Furthermore, we have identified which specific strains have predispositions for increased adiposity accumulation, total weight, circulating analyte levels, and metabolic phenotypes.

Like the CC, the relationship between weight and adiposity is not always uniform within humans (Hashimoto et al., 2016; Verheggen et al., 2016). While the CC mostly showed a strong positive relationship between adiposity and weight, several strains such as CC011/Unc, CC008/GeniUnc, and CC059/TauUnc that weighed more than the majority of other strains had only ~15% body fat, compared to the fattest strains with 20–23% body fat. Similar to the relationship between weight and adiposity, the relationship between adiposity and overall metabolic health can vary within humans (Yaghootkar et al., 2014, 2016; Ding et al., 2016; Gonçalves et al., 2016; Iacobini et al., 2019). At both baseline and post-diet, significant associations between body fat % and individual markers of metabolic health were only detected consistently for body fat % and insulin, and body fat % and alanine transaminase (ALT). One possible explanation for the lack of associations obtained is the nocturnal eating pattern of mice, since the concentration of glucose and insulin fluctuates with their circadian rhythms (Jensen et al., 2013), though the number of hours that the mice were fasted prior to the blood draw could also have minor effects on the analytes measured.

By estimating the average metabolic health of each CC strain via calculation of a metabolic health score, we identified CC028/GeniUnc and CC040/TauUnc as two of the fattest strains in our study that were healthier than the leaner strains CC030/GeniUnc and CC041/TauUnc at baseline, whose body fat % were half of CC028/GeniUnc and CC040/TauUnc, mirroring the “sub-phenotypes” within obesity of metabolically “healthy” or “unhealthy” individuals found in human studies (Peppa et al., 2013; Dobson et al., 2015; Schulze, 2019). After the 8-week diet challenge, CC028/GeniUnc and CC040/TauUnc remained healthier than CC030/GeniUnc, while CC041/TauUnc was both leaner and healthier than these three strains, reflecting the strain-dependent effect of diet.

At baseline the body fat % measured in the CC mice demonstrated that the predisposition to developing obesity occurred in a strain-dependent manner; baseline body fat % also highlighted the wide phenotypic variation across strains and minor phenotypic variation within strains, which varied by trait and strain. For most traits at baseline such as total weight, TG, cholesterol, and glucose, the ranges of strain coefficients of variation (CV%) were within ~20%; for example, the CV% of total weight for each strain was 4.91–23.2% where CC030/GeniUnc exhibited the lowest within-strain phenotypic variation (CV% = 4.91) and CC040/TauUnc exhibited the highest within-strain phenotypic variation (CV% = 23.2%). The range of strain CV% for baseline body fat % was slightly larger (13.9–44.7%), demonstrating that certain traits may be more sensitive to environmental differences such as being housed in different cages which could lead to differences in microbiome exposure, or minor genetic differences since completed CC lines are at least 98% homozygous (UNC Systems Genetics Core Facility, 2012) but not necessarily the same degree of homozygosity across individuals.

By analyzing the post-diet metabolic traits measured in these 22 CC strains together, our data recapitulates some key findings in humans by Sims (1976). As expected, metabolic rate estimated as heat production had the strongest inverse relationship with post-diet body fat %, which implies that body fat % increases as metabolic rate decreases. Body fat % was not significantly correlated with basal activity, showing that spontaneous physical activity alone did not significantly alter the degree of adiposity accumulation. Remarkably, energy intake decreased as body fat % increased; when adjusted for total body weight, this negative correlation increased in both strength and significance regardless of diet (Supplementary Figure 8), suggesting that the body attempts to adjust energy consumption and maintain energy homeostasis when adiposity is in excess, as reflected by changes in hormone levels that regulate energy consumption such as increased leptin secretion from adipose tissue (Caro et al., 1996; Friedman and Halaas, 1998) and lower levels of the gut satiety-related peptide tyrosine-tyrosine (PYY) found in obese individuals (Simpson et al., 2011). As body fat % increases, the secretion of the satiety hormone leptin from adipocytes also increases, which would lead to a decrease in appetite and therefore a decrease in feed consumption. Because the HS diet contains 290 g of sucrose for 1,042.8 g of HS diet and the HP diet contains 113 g of sucrose for 1,000.1 g of HP diet, another potential explanation for the negative correlation between energy intake and body fat % is the glucostatic theory, which states that glucose availability and utilization in specific regions of the brain may affect the regulation of satiety perception and short-term regulation of appetite (Mayer, 1953). Thus, for two mice consuming the same grams of experimental diet, the mouse fed the HS diet would consume more sucrose than the mouse fed the HP diet, resulting in a difference in the availability of glucose for each mouse and possible differences in the utilization of macronutrients depending on the strain (genetic effects). For example, the night RER of CC002/Unc was 0.847 on the HP diet and 0.921 on the HS diet, whereas the night RER of CC008/GeniUnc was 0.821 on the HP diet and 0.824 on the HS diet (Supplementary Table 10). Future studies using isocaloric diets with more extreme differences only in fat content or only sucrose content would help determine whether the stronger negative correlation between energy intake after correcting for total weight and body fat % of mice fed the HS diet is attributed to increased fat or sucrose content.

As accumulation of adiposity increased, RER decreased which implies increased utilization of fat as the substrate for energy expenditure since fat is in excess. RER was strongly positively correlated with heat production, illustrating that the increase in metabolic rate shifts substrate utilization toward carbohydrates and away from fat. If energy expenditure remains unchanged, the metabolic flexibility of shifting from carbohydrate utilization toward lipid utilization would compensate for the decrease in energy consumption (Farias et al., 2011; Goodpaster and Sparks, 2017). Along with the strong positive correlation between heat production and energy intake, the relationships between metabolic traits reaffirm the implication of energy balance. The consistency between the current results and Sims' results demonstrates the ability of the CC to reliably model human genotypic and phenotypic variation when studying complex traits.

After 8 weeks of feeding the CC mice either the HP or HS diet, assessing body composition in the CC revealed the strains' different responses to diet in terms of weight gain and other phenotypic changes in obesity-related traits. Consistent with the findings of Barrington et al. (2017), the strength of the effect of diet depended on the trait examined, macronutrient composition, and subject strain (genetic background). For example, certain CC strains did not respond to differences in macronutrient composition, either remaining persistently fat (CC040/TauUnc, CC063/Unc, CC001/Unc) or lean (CC019/TauUnc) regardless of experimental diet, while other strains clearly accumulated less body fat % on the HP diet compared to the HS diet (CC028/GeniUnc, CC004/TauUnc, CC006/TauUnc). Furthermore, experimental diet alone did not have a significant effect in general on circulating glucose, insulin, nor TG based on the results of the linear mixed model analysis, but certain CC strains showed drastic differences in phenotypic response to diet for these traits, such as CC036/Unc, CC002, and CC004/TauUnc for TG; CC036/Unc and CC040/TauUnc for glucose; and CC040/TauUnc, CC004/TauUnc, CC045/GeniUnc, and CC032/GeniUnc for insulin. The different response to diet by CC strain suggests that variation in genetic architecture may contribute to differences in individual nutrient need and substrate utilization, which should be taken into account when developing weight loss strategies.

Similar to the findings in this study, a recent large-scale human study performed by Berry et al. (2020) examining postprandial metabolic response to food relative to precision nutrition highlighted large inter-individual variability when subjects were fed identical meals, and found that genetic background and environmental factors, including person-specific factors (e.g., the microbiome) and meal macronutrients, had varying degrees of influence on traits assessed. Mirroring the broad range of phenotypic response to diet in the CC, human participants in the DIETFITS Randomized Clinical Trial that were administered either a low-fat or low-carbohydrate diet also exhibited a wide range of response to diet in terms of weight loss over 12 months, regardless of their genotypes defined by three SNPs (Gardner et al., 2018). Due to the complex etiology of obesity, studies in humans endeavoring to prove direct relationships between individual SNPs and obesity have succeeded in finding associations between genetic loci and body weight (Deeb et al., 1998; Scuteri et al., 2007; Speliotes et al., 2010; Claussnitzer et al., 2015; Hägg et al., 2015), but translational application of these associations will first require further investigation into the biological function of novel obesity-associated genetic loci (Loos, 2018) and elucidation of the causes behind conflicting findings where associations between genetic loci and phenotypes were not detected (Sørensen et al., 2006; Drabsch et al., 2018; Gardner et al., 2018; Merino et al., 2018). Nevertheless, the phenotypic variation in adiposity by CC strain in this study clearly illustrate the genetic predisposition for developing obesity, concurrent with findings in humans (Stunkard et al., 1986; Bouchard and Tremblay, 1997; Viitasalo et al., 2019). Therefore, effective mitigation of obesity using personalized nutrition would ideally incorporate information regarding an individual's genetic background, behavior, environmental influences, physiological response to diet, and socioeconomic situation in addition to their genotype in terms of recommendations for alterations in diet and exercise levels (Drabsch and Holzapfel, 2019).

One caveat of our study design is that we cannot assess the effect of aging nor whether there are strain specific age-related phenotypes given the natural variation both between strains and between individuals within strains. Similar to the current study, a preprint of a pending manuscript utilizing B × D mice indicates that certain age-related phenotypes such as longevity and weight are under strong genetic regulation and are also affected by diet and gene-by-environmental interactions (Roy et al., 2019). Our diet challenge and age are confounded and we cannot assess differences in genetic susceptibility that are age dependent. Additional investigations using a modified study design could effectively assess the effect of aging on metabolic factors in CC mice.

Although basal activity levels were assessed, one limitation of this study is the lack of “true” exercise activity (e.g., wheel running), since increased weight loss in humans has been shown to be associated with increased physical activity if calorie intake is controlled (Zemel et al., 2009). Another caveat of this study is the unavailability of metabolic phenotype data for the mice at baseline (e.g., energy expenditure, feed intake, RER, basal activity), which limits the conclusions that can be made regarding the effects of diet compared to feed intake on energy balance when interpreting these data. Moreover, recent studies have found that the gut microbiota also potentially play a significant role in the development of obesity (Tilg and Kaser, 2011; Pace and Crowe, 2016; Lee et al., 2018). Further studies should be performed with multiple genetically diverse populations to determine which diets would be most effective for weight loss by individuals according to their genetic background and to examine the state of epigenetic markers and transcript expression levels in specific tissues.

## Data Availability Statement

Raw metabolomics data are available at the NIH Common Fund's National Metabolomics Data Repository (NMDR) website, the Metabolomics Workbench, https://www.metabolomicsworkbench.org, where it has been assigned Project ID PR001032. The data can be accessed directly via its Project doi: 10.21228/M89D63.

## Ethics Statement

The animal study was reviewed and approved by University of North Carolina Institution Animal Care and Use Committee.

## Author Contributions

PY assisted with data collection, analyzed the data, performed the statistical analysis, and drafted the manuscript. JA and MV maintained the mice, performed the experiment on them, and collected data. EG also collected and processed data. BB conceived of and designed the study with the assistance of FP-M. BB performed analysis and interpretation of the data, and drafted the manuscript. All authors contributed to the article and approved the submitted version.

## Conflict of Interest

The authors declare that the research was conducted in the absence of any commercial or financial relationships that could be construed as a potential conflict of interest.

## Supplementary Material

Supplementary File 1 All supplementary figures.

Supplementary File 2 All supplementary tables.

Supplementary File 3 R code, data required for generating baseline figures and calculating baseline H^2^ estimates, and a README file with brief descriptions of all contents within Supplementary File 3.

Supplementary File 4 R code, data required for generating post-diet figures and calculating post-diet H^2^ estimates, and a README file with brief descriptions of all contents within Supplementary File 4.
