# Supplementary material for: Genetic Background Shapes Phenotypic Response to Diet for Adiposity in the Collaborative Cross
Source: Front Genet. 2021 Feb 11;11:615012. doi: 10.3389/fgene.2020.615012 (PMC7905354; doi:10.3389/fgene.2020.615012)
Supplement: Supplementary File 1 — All supplementary figures. [file Data_Sheet_1.docx]

**SUPPLEMENTARY METHODS AND FIGURES**

**Supplementary methods for Liquid chromatography–mass spectrometry (LC-MS)**

***Sample processing.***

Briefly, samples (20 μl plasma) were aliquoted to a 2 ml Eppendorf tube and mixed with 80 μl of 5 μM surrogate standard. Samples were vortexed for 30 seconds and centrifuged at 18,000 g at 10°C for 10 min. Supernatant was transferred to 150 μl glass inserts in High Performance Liquid Chromatography (HPLC) vials. To prepare standards for sample quantification, 80 μl of 5 μM SSTD and 20 μl of each standard were aliquoted directly to the glass inserts in HPLC vials and briefly vortexed.

***LC/MS/MS.***

Supernatants (5 μl) were analyzed by injection onto a silica column (150 by 2 mm, 3 um particle Silica (2) with 100 Angstrom; Catalog #00F-41620-B0, Phenomenex, Torrance, CA) at a flow rate of 0.25 ml/min using a Waters Acquity UPLC (Waters, Milford, MA) interfaced with an API 4000 Q-TRAP mass spectrometer (AB SCIEX, Framingham, MA). A discontinuous gradient was generated to resolve the analytes by mixing solvent A (0.1% acetic acid in water) with solvent B (0.1% acetic acid in methanol) at different ratios starting from 2% B linearly to 15% B over 5 min, then linearly to 100% B to 6.25 min, then hold to 8 min, and then back to 2% B at 6.25 min and held until 10 min.

Analytes were monitored using electrospray ionization in positive-ion mode with multiple reaction monitoring (MRM) of precursor and characteristic production transitions as shown in Table S3. The parameters for the ion monitoring were as follows: spray voltage, 4.5 kV; curtain gas, 15; GS1, 60; GS2, 50; CAD gas, medium; Nitrogen (99.95% purity) was used as the source and collision gas.

***Precision and accuracy.***

Three different human plasma sample controls with known concentrations were run in triplicate extraction for each sample batch of less than 60 samples for quality control and to establish the intraassay coefficient of variation (CV). The interassay CV was determined by assaying aliquots of these pooled samples for each of the 9 days that a batch of samples LC-MS was performed. Analyte concentrations from the batch were acceptable when the accuracy of the values determined from each QC sample were within 100 ± 10% of their expected values and the intrabatch CVs for the same QC samples were all less than 10%. Integration and quantification of values was done using Analyst 1.6.2 software (AB SCIEX, Singapore). Standard linearity was calculated using linear regression model.


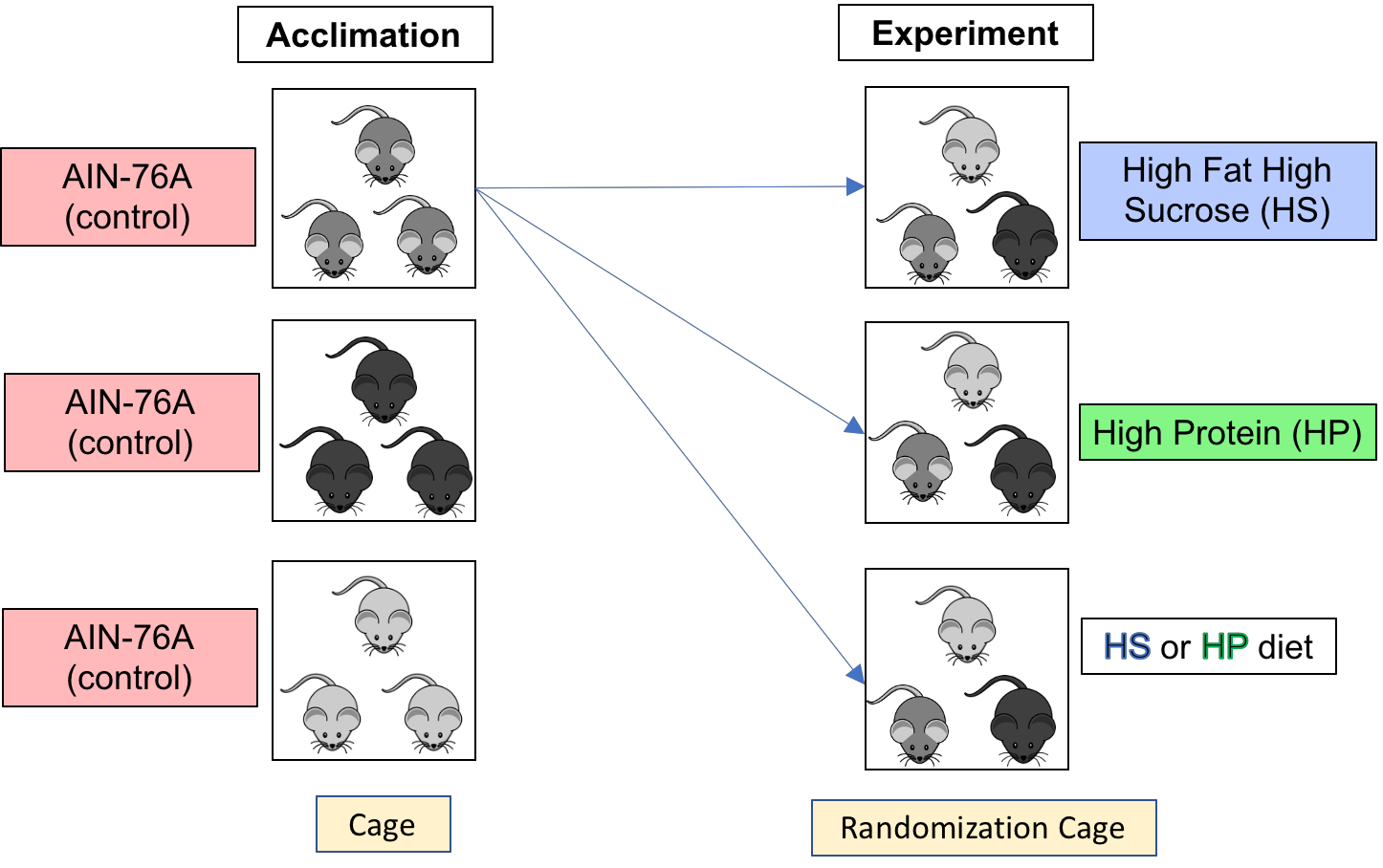


**Supplementary Figure 1. Mouse randomization procedure**

During the 2-week acclimation phase, all mice were fed the AIN-76A synthetic chow diet and housed by strain, one strain per cage. Prior to the experimental diet challenge, the mice were randomly assigned to either the high fat high sucrose (HS) or high protein (HP) diet, and subsequently the strain siblings were moved to new cages according to the assigned experimental diet. Mice were housed according to experimental diet for the duration of the 8-week diet challenge.


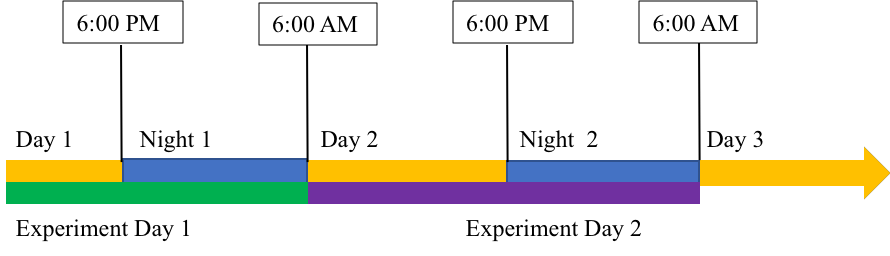


**Supplementary Figure 2. PhenoMaster TSE assessment 12-hr light/dark cycles**

Indirect calorimetry was assessed over three days using PhenoMaster (TSE Systems) automated home cage phenotyping. Dark cycles began at 6:00 PM and ended at 6:00 AM, and complete light cycles began at 6:00 AM and ended at 6:00 PM. Means of each phenotype measured were calculated for individual light cycles by day (day 1, 2, and 3), for individual dark cycles by day (night 1 and 2), for light cycles for the duration of the experiment (all days combined), for dark cycles for the duration of the experiment (both nights combined), and for individual experiment days (experiment day 1 and 2). Measurements were taken for less than 12 hours during the light cycles at the beginning and end of the experiment (days 1 and 3), while measurements for both dark cycles (nights 1 and 2) and the light cycle between (day 2) for 12 complete hours.


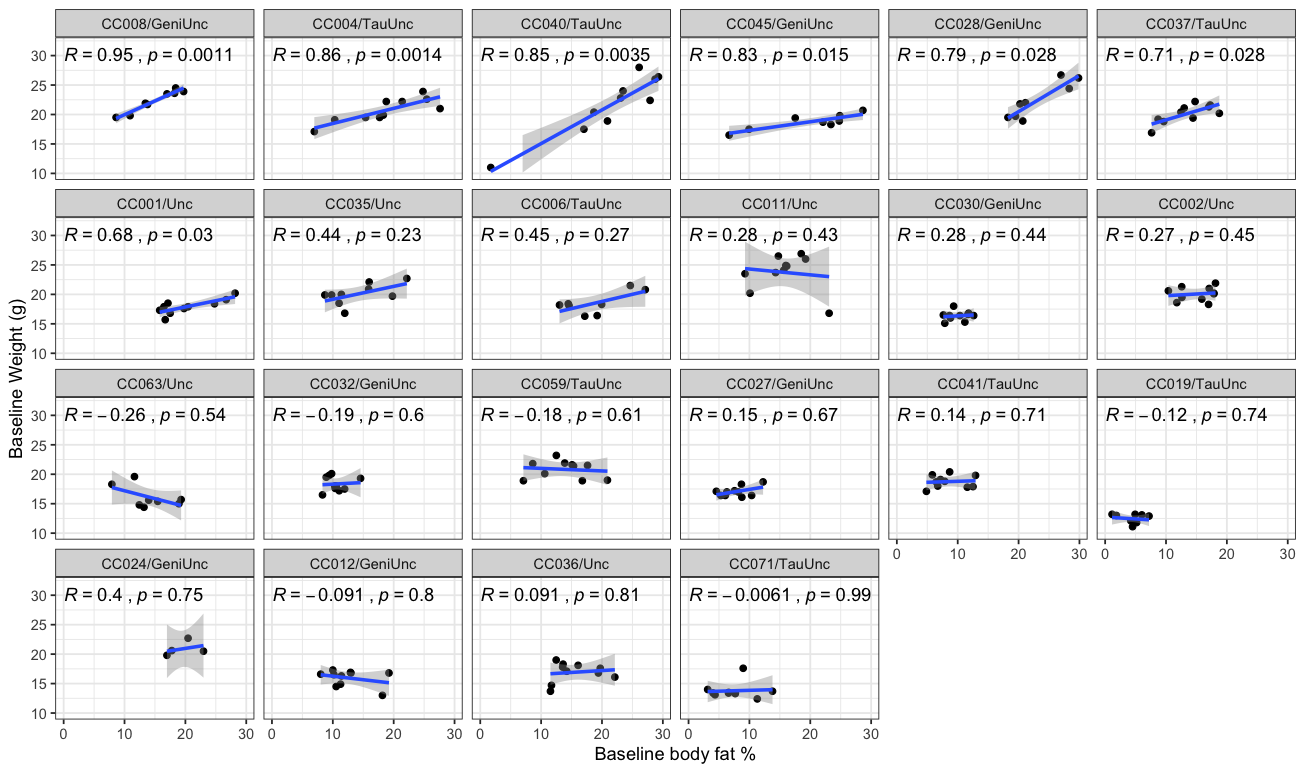


**Supplementary Figure 3. Spearman’s correlation of baseline body fat % and total weight (g)**

Spearman’s correlation of baseline body fat % and total weight (g) was performed for each CC strain to estimate whether the relationship between body fat % and weight differed by genetic background. Strains are ordered by significance of correlation from most to least significant.

**
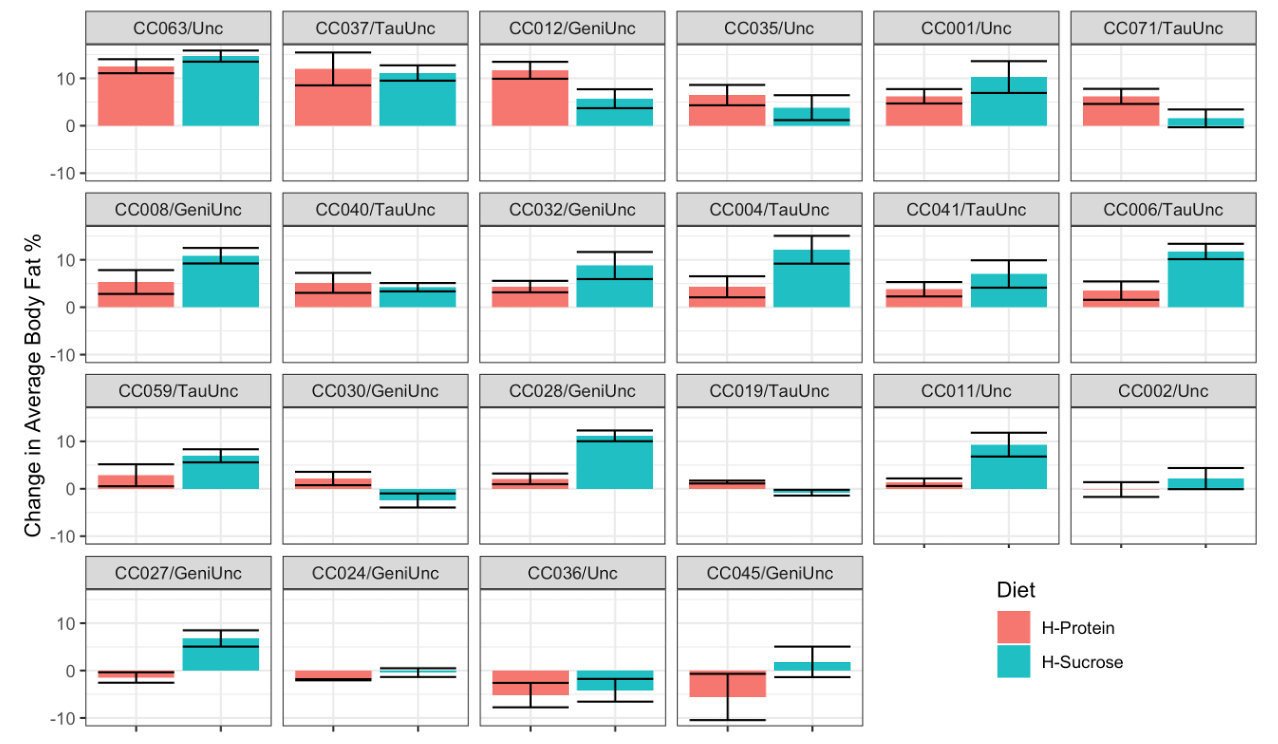
**

**Supplementary Figure 4. Average changes in body fat % for each CC strain on the HP or HS diet**

Changes in body fat % for each CC strain on the HP (H-Protein) or HS (H-Sucrose) diet were obtained by subtracting baseline body fat % from post-diet body fat % for each mouse, and then the mean change for each strain on the respective diets was calculated. Data are mean ± SE.

**
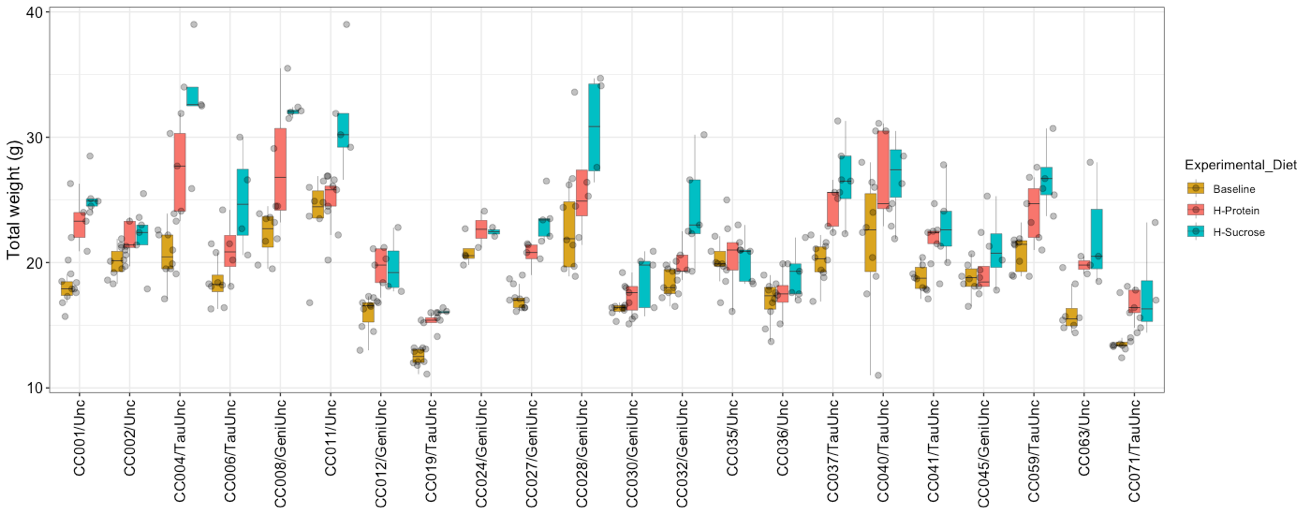

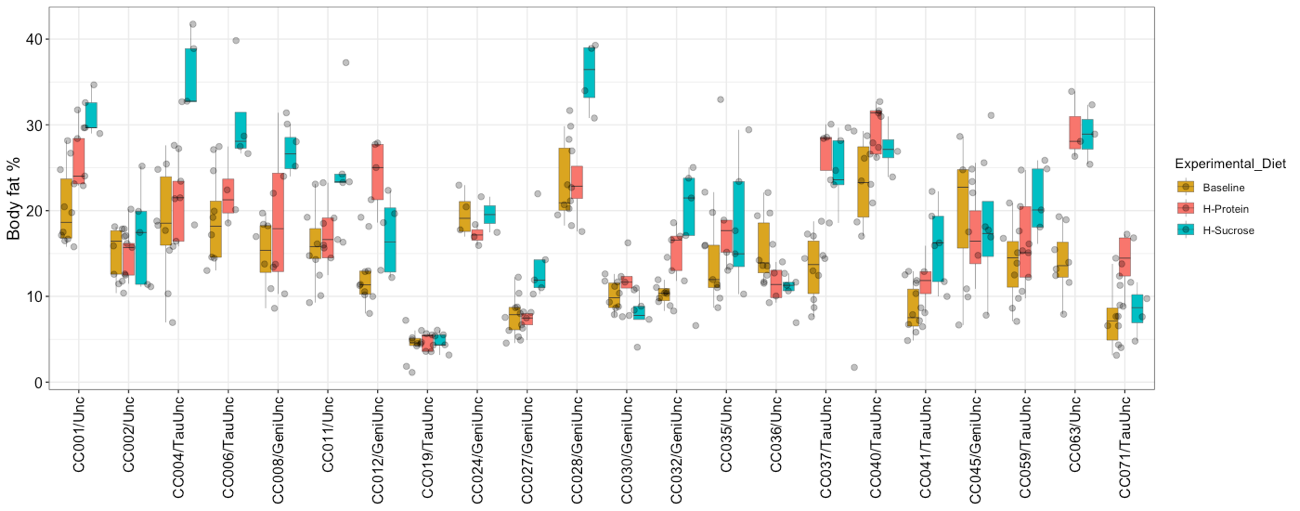
**A

B

C

**
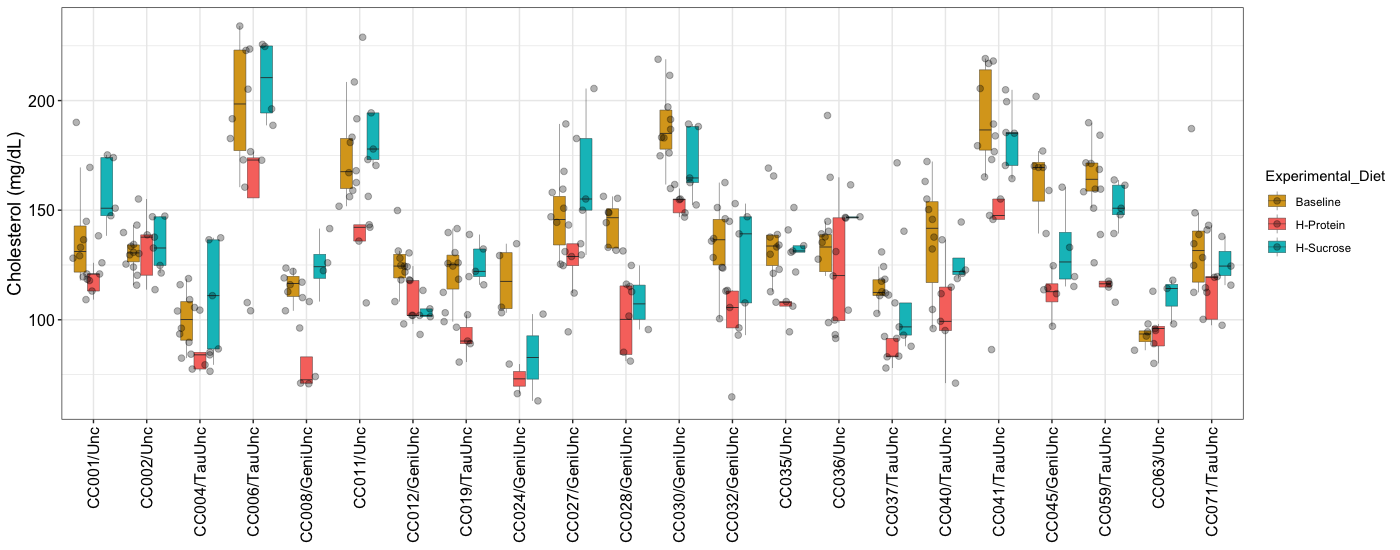
**

**Supplementary Figure 5.** **Baseline and post-diet measurements of body fat %, total weight, and cholesterol by strain and diet**

Baseline and post-diet measurements are shown for (A) body fat %, (B) total weight, and (C) total cholesterol to compare phenotypic differences between strain and diet. Strains are ordered numerically. H-Protein and H-Sucrose represent the HP and HS diets, respectively. Points are measurements obtained for each mouse.

A


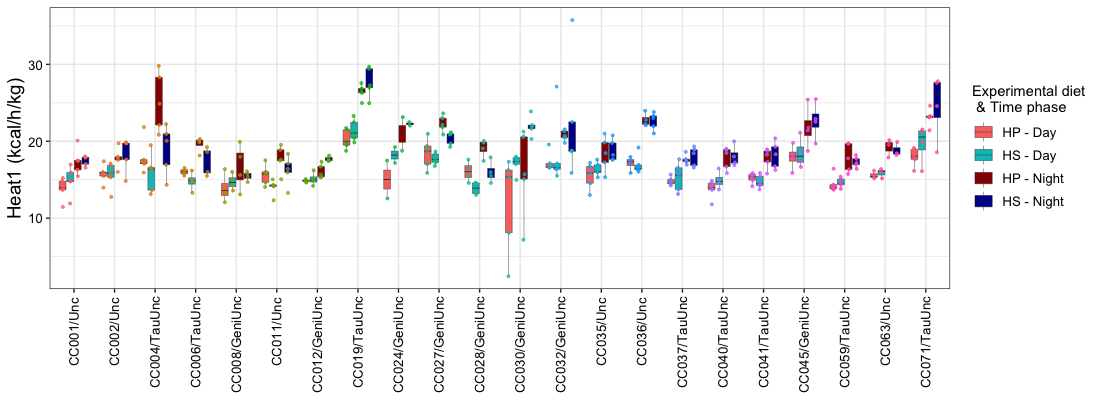


B


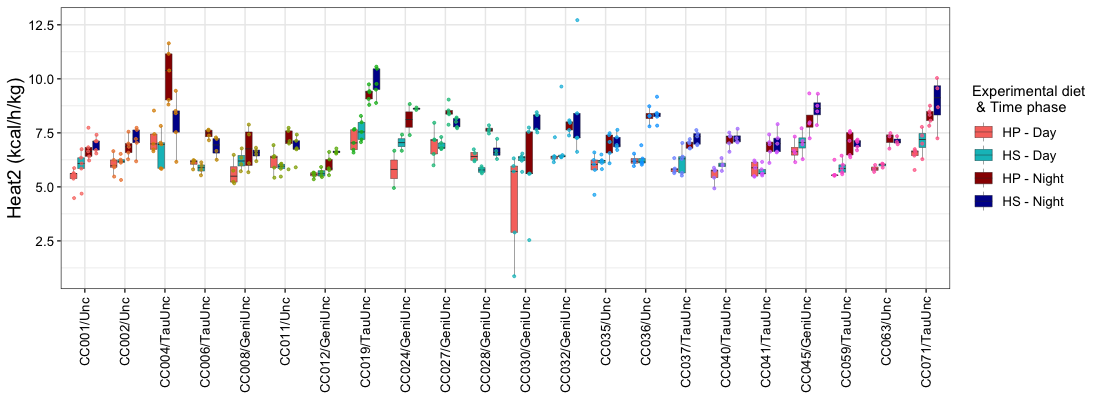


**Supplementary Figure 6. Average post-diet heat expenditure adjusted for total body mass (Heat1) and for lean mass only (Heat2)**

Post-diet quantification of average (A) heat expenditure adjusted for total body mass (kcal/h/kg) and (B) heat expenditure adjusted for lean mass (kcal/h/kg) for each CC strain on each diet show a wide range of variation across and within strains. Individual points are colored by strain. Data for each strain are ordered by diet within the day cycle followed by diet within the night cycle.

A


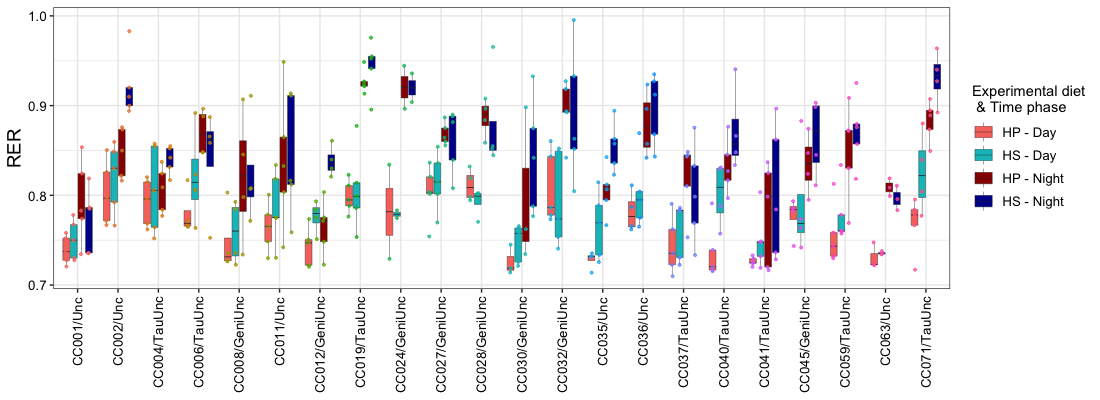


B
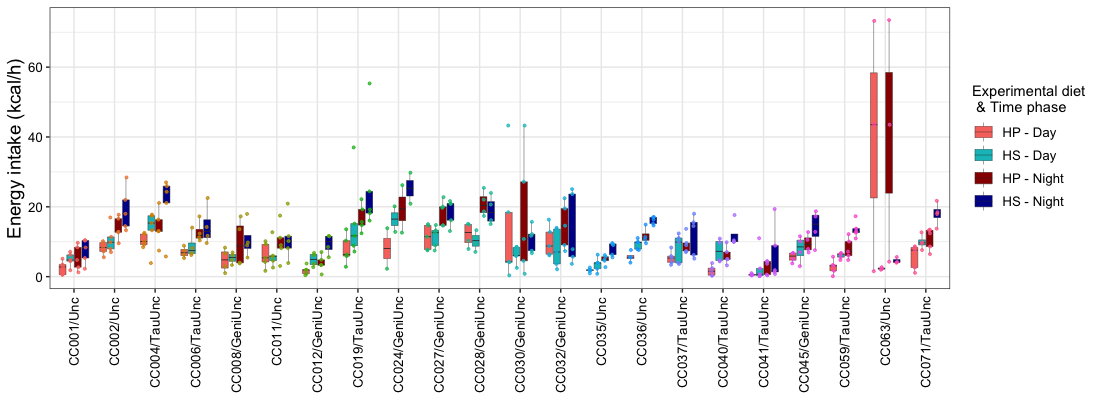


C


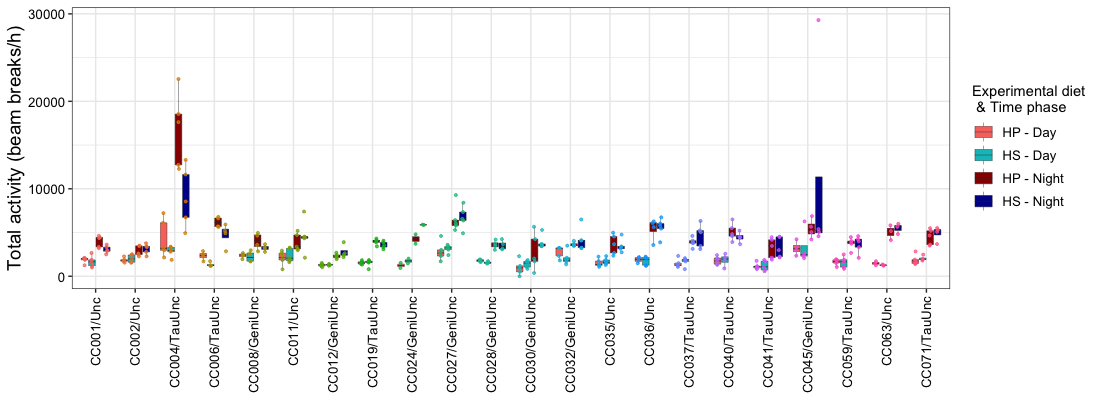


**Supplementary Figure 7. Average post-diet RER, energy intake, and total basal activity**

Post-diet quantification of average (A) RER, (B) energy intake (kcal/h), and (C) total basal activity (beam breaks/h) for each CC strain on each diet show a wide range of variation across and within strains. Individual points are colored by strain. Data for each strain are ordered by diet within the day cycle followed by diet within the night cycle.

**
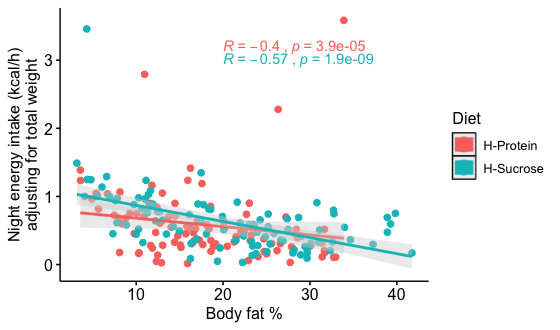
**

**Supplementary Figure 8. Spearman's correlation of post-diet body fat % and night energy intake adjusting for total body weight**

Spearman’s correlations between post-diet body fat % and nocturnal energy intake adjusting for total weight for the HP (rho=-0.4, p=3.9x10^-5^) and HS (rho=-0.57, p=1.9x10^-9^) diets. H-Protein and H-Sucrose represent the HP and HS diets, respectively. R indicate rho values.
